# Supplementary material for: Ecophysiology and interactions of a taurine-respiring bacterium in the mouse gut
Source: Nat Commun. 2023 Sep 18;14:5533. doi: 10.1038/s41467-023-41008-z (PMC10507020; doi:10.1038/s41467-023-41008-z)
Supplement: Supplementary file 1 — Supplementary Information [file 41467_2023_41008_MOESM1_ESM.pdf]

Supplementary Information

Ecophysiology and interactions of a taurine-respiring bacterium in the mouse gut

Huimin Ye, Sabrina Borusak, Claudia Eberl, Julia Krasenbrink, Anna S. Weiss, Songcan Chen, Buck T. Hanson, Bela Hausmann, Craig W. Herbold, Manuel Pristner, Benjamin Zwirzitz, Benedikt Warth, Petra Pjevac, David Schleheck, Bärbel Stecher, and Alexander Loy

Content

Supplementary Text

Methods

Results & Discussion

Supplementary Figures

Supplementary References

Supplementary Text

Methods

Enrichment and isolation of strain LT0009

Intestinal content (cecum and colon) of wild-type C57BL/6 mice co-housed with access to normal chow and water *ad libitum* was used as inoculum for the enrichment cultures. Gut content was collected immediately after sacrifice of the mice in an anaerobic chamber and homogenized in 50 ml sterile, anoxic 30 mmol/l bicarbonate buffer (pH 7) for inoculation. A modified *Desulfovibrio* medium (based on DSMZ medium 641, www.dsmz.de) in which all sulfur-containing chemicals were omitted (i.e., Na_2SO_4, $Na_2S_2O_3$, $MgSO_4$, and Na_2S) was used for enrichment of taurine-respiring microorganisms in presence of lactate and pyruvate as electron donors. The basal medium consisted of (per liter of final medium) 1 g NH_4Cl, 2.1 g Na_2Cl, 0.825 g $MgCl_2$ x $6H_2O$, 0.1 g $CaCl_2$ x $2H_2O$, 0.5 g KH_2PO_4, 1 g yeast extract (Thermo Fisher Scientific, US), 1 mL trace element solution SL-10 (DSMZ medium 320), and 1 ml selenite-tungstate solution (DSMZ medium 385). The basal medium was autoclaved, supplemented with 0.2 µm filter-sterilized $NaHCO_3$ stock solution (2 g $NaHCO_3$ per liter of final medium) and 10 ml vitamin solution (DSMZ medium 141), and placed in an anaerobic chamber (Coy labs, USA) under anoxic atmosphere (85% N_2, 10% CO_2, 5% H_2) for two days to ensure sufficient equilibration. All further work was done in the anaerobic chamber if not otherwise stated. The final modified *Desulfovibrio* medium was supplemented by (final concentrations) 10 mmol/l taurine, 10 mmol/l L-lactate, 20 mmol/l pyruvate, and 0.2 mg/l 1,4-naphthoquinone; each added from filter-sterilized stock solutions that were prepared in into autoclaved serum bottles and crimp sealed with sterile rubber stoppers and made anoxic by sparging with N_2 gas for 0.5 h. Choice of substrates and their concentrations was based on previous physiological experiments with *B. wadsworthia* strains [1–3]. Four ml of this final medium were distributed into sterile 20 ml Hungate tubes closed with sterile butyl rubber stoppers. Each tube was inoculated with one ml of supernatant of the homogenized intestinal content. The cultures were incubated at 37°C, periodically sampled for H_2S and taurine quantification, and sub-cultivated into new tubes by transfer of one ml into four ml fresh media every two to three days over 8 weeks. Isolation of enriched taurine-metabolizing, sulfidogenic microorganisms was performed using differential agar plates in the anaerobic chamber. The plates contained the basal medium supplemented with 2.5 g/l Na-L-lactate, 1.25 g/l taurine, 2.2 g/l sodium pyruvate, 200 µg/l 1,4-naphthoquinone, and 0.5 g/l ammonium ferric citrate and were solidified with 1.5% agar; the H_2S produced from taurine and the ferric iron form a black iron sulfide precipitate. Black colonies were picked and streaked onto fresh plates until a uniform colony morphology was observed. Single colonies were inoculated back into 5 ml liquid

medium and these cultures were further purified by dilution to extinction, yielding strain LT0009 as an isolate. The isolation process was monitored by microscopy and direct Sanger sequencing of bacterial 16S rRNA gene amplicons. The purity of the culture was additionally confirmed by fluorescence *in situ* hybridization (FISH) using a newly developed LT0009-specific probe. The isolate was stored in 5% DMSO (1 ml culture plus 1 ml 10% DMSO in sterile water at -80°C.

Growth experiments with strain LT0009

Strain LT0009 was routinely grown at 37°C in 5 ml modified *Desulfovibrio* medium described above in 20 ml Hungate tubes under an atmosphere of 10% CO_2, 5% H_2, and 85% N_2 and closed with butyl rubber stoppers. Substrate utilization tests were also performed in 96-well plates (transparent, F-bottom, Greiner bio-one cellstar) in the anaerobic chamber. During these incubations, optical density at 600 nm (OD_{600nm}) was measured every 30 minutes in a plate reader (Multiskan Go, Thermo Scientific) with 5 s shaking prior to measurements.

To determine the optimal taurine concentration for LT0009 growth, different taurine concentrations (10, 20, 40, 60, 80, and 100 mmol/l, each in triplicate) in 200 µl of the modified *Desulfovibrio* medium, amended with 10 mmol/l lactate, 20 mmol/l pyruvate, and 0.2 mg/l 1,4-naphthoquinone, were used for inoculation of 40 µl of LT0009 culture. Growth was measured with the plate reader. Growth of LT0009 in 10 mmol/l taurine was additionally tested with the same setting but without pyruvate.

For substrate utilization tests, the modified *Desulfovibrio* medium was supplemented with different electron donors with taurine as the electron acceptor. Tested substrate combinations included: lactate/taurine, formate/taurine, pyruvate/taurine, hydrogen/acetate/taurine. Fermentative growth with pyruvate or taurine in absence of a dedicated electron donor was also tested. All cultures were amended with 10 mmol/l of each substrate and 1,4-naphthoquinone at 0.2 mg/l. Hydrogen was flushed into Hungate tubes to a pressure of two bars.

Utilization of different organic and inorganic sulfur compounds (each 10 mmol/l) as electron acceptors was tested in modified *Desulfovibrio* medium with a final concentration of 10 mmol/l lactate, 20 mmol/l pyruvate, and 0.2 mg/l 1,4-naphthoquinone. The sulfur compounds included: taurine（Sigma-Aldrich, cat.no. T8691), sodium sulfate (Carl Roth, cat.no. 8560.3), sodium sulfite (Sigma-Aldrich, cat.no. 71922), racemic sulfolactate (synthesized as described previously [4]), sodium thiosulfate (Sigma-Aldrich, cat.no. 217263), L-cysteate (Sigma-Aldrich, cat.no. 30170), racemic 2,3-dihydroxypropane-1-sulfonate (DHPS) (synthesized as described previously [5]), and isethionate (Sigma-Aldrich, cat.no. 820708010). 100 µl of LT0009 culture was transferred into triplicate tubes to test the growth with each sulfur compound. For differential proteomics and transcriptomics, cells of LT0009 were harvested from cultures grown with taurine, sulfolactate, or thiosulfate in the late exponential growth phase.

The pH range for optimal growth was assessed at pH 4.0, 5.0, 6.0, 6.5, 7.0, 7.5, 7.9, and 8.5 at 37°C in triplicate Hungate tubes. The pH was adjusted with 0.1 mol/l HCl or 1 mol/l NaOH followed by filter sterilization. The temperature range for optimal growth was tested at 20, 27, 30, 32, 37, 42, 61, and 75°C in triplicate at pH 7.2. OD at 600 nm was measured periodically during growth using a spectrophotometer (Ultrospec 10, Amersham Bioscience). LT0009 was incubated at 37°C on modified *Desulfovibrio* medium agar plates under aerobic conditions to test its susceptibility to oxygen.

Glycerol cryo-stocks of a strain mixture of the synthetic, 12-member Oligo-Mouse-Microbiota (OMM^{12}) were grown in Anaerobic Akkermansia Medium (AAM) in an anaerobic chamber (90% N_2, 5% CO_2, 5% H_2) at 37°C for 24 h. The OMM^{12} community consists of *Acutalibacter muris* KB18, *Akkermansia muciniphila* YL44, *Bacteroides caecimuris* I48, *Bifidobacterium animalis* YL2, *Blautia coccoides* YL58, *Enterocloster clostridioformis* YL32, *Clostridium innocuum* I46, *Enterococcus faecalis* KB1, *Flavonifractor plautii* YL31, *Limosilactobacillus reuteri* I49, *Muribaculum intestinale* YL27, and *Turicimonas muris* YL45. Subcultures were transferred into fresh AAM with and without 10 mM taurocholic acid (TCA, Carl Roth, cat.no. 8149.3) and grown for 24 hours. OMM^{12} spent media were produced by filter-sterilization (0.22 µm) of supernatants from these cultures. LT0009 was subsequently grown in these two OMM^{12} spent media produced with or without TCA to investigate if growth of LT0009 depends on taurine release by deconjugation of TCA by OMM^{12} strains. As additional controls, LT0009 was grown in OMM^{12} spent medium produced without TCA but subsequently supplemented with taurine (10 mM) or TCA (10 mM). All cultures were amended with 10 mmol/l lactate, 20 mmol/l pyruvate, and 0.2 mg/l 1,4-naphthoquinone. OD at 600 nm was measured using a spectrophotometer (Ultrospec 10, Amersham Bioscience). Cultures were sampled at 0 h and 84 h after inoculation of LT0009 for metabolite quantification.

Substrate and metabolite quantification

For quantification of substrate removal and metabolite formation, strain LT0009 was grown in triplicate in 20 ml Hungate tubes with an initial concentration of 10 mmol/l taurine, 10 mmol/l lactate, 20 mmol/l pyruvate, and 0.2 mg/l 1,4-naphthoquinone. The culture was subsampled at time points 0, 24, 40, 48, 64, 72, 88, and 98 h for OD and substrate/metabolite quantification. Taurine in cell-free samples was quantified using 4-fluoro-7-nitrobenzofurazan as a derivatizing agent as previously described [6] using an Infinite 200 PRO spectrophotometric microplate reader (TECAN Group Ltd., Männedorf, Switzerland) with excitation set at 470 nm and emission set at 530 nm. H_2S in cell-free samples was quantified spectrophotometrically as previously described [7] with absorbance measurements at 670 nm using an Infinite 200 PRO spectrophotometric microplate reader (TECAN Group Ltd., Männedorf, Switzerland). Dilutions of a sodium sulfide solution (Na_2S; Sigma-Aldrich, cat.no. 71988) were used as external standards for H_2S quantification. Short-chain fatty acids (SCFA) were measured using P/ACE-MDQ capillary

electrophoresis equipped with an UV-detector (Beckman Instruments, Krefeld, Germany). Samples were diluted 1:20 with a working solution consisting of 0.01 mol/l NaOH, 0.5 mmol/l $CaCl_2$, and 0.1 mmol/l caproate (internal standard). A dilution series of a stock mixture of sulfate, formate, succinate, acetate, lactate, propionate, butyrate, and valerate (1 mmol/l each) was used as external SCFA standards for quantification. CEofixTM Anions 5 kit (Analis, Belgium) was used for SCFA measurement according to the manufacturer's instruction.

Electron and fluorescence microscopy

For scanning electron microscopy, 5 ml of cells were prefixed with buffered glutaraldehyde (2.5% v/v) and 10 µl of prefixed cells were spotted onto poly-L-lysine coated glass slides (Corning BioCoat, USA). The glass slides were dried at room temperature and washed three times in 0.1 cacodylate and 5 g/l sucrose buffer. Washed slides were postfixed in a 1% (w/v) osmium solution for 40 min and rewashed three times with the cacodylate sucrose buffer. Subsequently, the slides were dehydrated in an ethanol series (30, 50, 70, 90, 96, and 100% ethanol in distilled water) for 5 min each, followed by two additional washing steps in 100% ethanol. After dehydration, the slides were dried with 100% ethanol using a Critical Point Dryer 300 instrument (Leica), mounted onto stubs, and gold sputter-coated (sputter coater JFC-2300HR, JOEL). Images were obtained with a scanning electron microscope (JSM-IT300, JOEL).

Paraformaldehyde fixation of LT0009 cells from an actively growing culture, fluorescence *in situ* hybridization (FISH) with 5'-end-mono-labeled rRNA-targeted probes (Biomers, Ulm, Germany) (Supplementary Data 2), and counter-staining with 4'-6-diamidino-2-phenylindole (DAPI) was performed as previously described [8]. A mouse colon tissue section was obtained from a previous study [9]. Cells and the tissue section were imaged using a confocal laser scanning microscope (Leica TCS SP8X, Germany) and FISH pictures were analyzed using the image analysis software daime [10]. A new probe TAU1151 was designed for LT0009 and related 16S rRNA sequences using the SILVA NR99, release 132 16S rRNA database [11], and probe design tools of the ARB program [12]. The hybridization buffer formamide concentration for optimal specificity and sensitivity of probe TAU1151 was determined by melting curve analysis (Supplementary Fig. 2). Probe MAIL1151 was designed as a competitor probe to eliminate nonspecific binding of TAU1151 to closely related *Mailhella* species. The SILVA database SSU_r138.1_REG was utilized to evaluate perfect-match coverage of the designed FISH probes using TestProbe 3.0 [13]. For increased specificity, MAIL1151 and TAU1151 should be used with different fluorophores and simultaneously in equimolar concentration.

Genome sequencing and annotation

Genomic DNA of strain LT0009 was extracted using the Wizard Genomic DNA purification Kit (Promega, USA) according to the manufacturer's procedures for both HiSeqV4 PE125 (Illumina) and MinION

sequencing (Oxford Nanopore Technologies, Oxford, UK). Illumina and Nanopore sequences were demultiplexed, followed by adapter and barcode trimming using qCAT v. 1.1.0 (https://github.com/nanoporetech/qcat). A hybrid assembly of both Illumina and Nanopore reads was performed using Unicycler v. 0.4.6 [14]. The genome was annotated using the MicroScope annotation platform [15], and genes of interest were manually curated using the tools integrated into the MicroScope annotation platform (https://mage.genoscope.cns.fr/) as described previously [16]. Briefly, proteins annotated as homologous to proteins with a known function had an amino acid identity ≥40% (over ≥80% of sequence coverage) to a Swiss-Prot [17] protein or manually curated protein. Proteins annotated as putative homologs of the respective database entries had an amino acid identity ≥25% (over ≥80% of sequence coverage) to a Swiss-Prot or TrEMBL [18] entry. Hydrogenase genes were detected by the MicroScope annotation platform and further classified into subgroups using the HydDB database tool [19].

Phylogenetic and phylogenomic analyses

The full-length 16S rRNA gene was retrieved from the genome of LT0009. Related 16S rRNA gene sequences with ≥80% similarity to LT0009 were recovered from the National Center for Biotechnology Information (NCBI) standard nucleotide database [20] and the SILVA database v.138 [13] using BLAST. 16S rRNA gene sequences of *Desulfovibrionaceae* type strains were extracted from List of Prokaryotic names with Standing in Nomenclature [21]. 16S rRNA gene sequences with ≥1,400 bp were dereplicated to remove redundant, 100% identical sequences, aligned using MUSCLE (v3.8.31) [22], and trimmed using TrimAl (v1.4. rev15) [23] with a gap threshold of 0.9. Maximum likelihood treeing of 16S rRNA gene sequences was performed using IQ-TREE (v. 1.6.2) [24] with 1,000x bootstrapping and model TVMe+R5. Sequence source environments were manually compiled from the NCBI SRA entries (Supplementary Data 7).

NCBI nucleic acid sequences with >60% coverage and >70% sequence similarity to *dsrAB* of LT0009 were recovered for phylogenetic analyses. Additional *dsrAB* sequences of related metagenome assembled genomes (MAGs) Mouse_MAG_UBA8003 (GCA_003512875.1) and *Mailhella massiliensis* strain Marseille-P3199 (GCA_900155525.1) were included in the phylogenetic analyses. Selected *dsrAB* nucleic acid sequences were aligned using MUSCLE (v3.8.31) [22] and trimmed using TrimAl (v1.4. rev15) [23] with -gt 0.1. The maximum likelihood tree was constructed using IQ-TREE (v1.6.2) [24] with 1000x bootstrapping [25] and automatic model selection.

Tpa, Xsc, and Ald homologs were identified using HMMscan (HMMER 3.3.2) [26] and the protein families database (Pfam A) [27], with the provided trusted cut-offs. Related amino acid sequences were collected from InterPro [28] databases using identifiers IPR005814 (Tpa), IPR017820 (Xsc), and IPR008141 (Ald). Amino acid sequences related (>40% coverage, >70% sequence identity) to the *dsrE*-like gene (TAU_v1_1364) and rhodanese-like gene *sbdP* (TAU_v1_1430) were identified by BLAST and retrieved from NCBI for

phylogenetic analyses. Amino acid sequences for *dsrEFH* genes (TAU_v1_1695, TAU_v1_1696, and TAU_v1_1697) were identified by hits to TIGRfam [29] and EggNOG [30] using HMM with e-value cutoff $1e^{-5}$. Additional DsrE, DsrEFH, and SbdP amino acid sequences were collected from the genomes in this study (Fig. 1b) using blastP with a minimum bit score of 100. CysH amino acid sequences were retrieved from NCBI using BLASTP (90% coverage and 65% sequence identity to CysH of *E. clostridioformis* YL32). Selected protein sequences were aligned with MAFFT (v7.475) [31], and the alignment was trimmed with TrimAl (v1.4.rev15) [23,31] with the flag -automated 1. Maximum-likelihood trees were created using the IQ-TREE web-server [32] with automatic substitution model selection and ultrafast bootstrapping (1000x) [25]. The trees were visualized with iTOL (version 6.7) [33].

Complete genome sequences of representative *Desulfovibrionaceae* strains were retrieved from NCBI and representative high-quality *Desulfovibrionaceae* MAGs were selected from the integrated mouse gut metagenome catalog (iMGMC) [34] for phylogenomic analyses. Phylogenomic treeing with the IQ-TREE ML method (v. 1.6.2, model: LG+R3 as chosen by automatic model and 1000 ultrafast bootstrap runs [25]) was based on 43 phylogenetic marker protein sequences that were aligned and concatenated using CheckM [35]. Average amino acid identity (AAI) and whole-genome average nucleotide identity (gANI) were calculated using the Enveomics Collection [36] and FastANI (v. 1.2) [37]. Genes involved in taurine (*tpa*), DHPS (*hpsGH*, *hpsO*, *hpsN*, *dphA*), 3-sulfolacetaldehyde (*slaB*), 3-sulfolactate (*suyAB*, *slsC*, *comC*), sulfoacetaldehyde (*xsc*, *sarD*), isethionate (*islAB*) and sulfite (*dsrABC*) metabolism were identified as described previously [38].

Presence of genes encoding thiosulfate reductases in LT0009 and *B. wadsworthia* genomes was revealed using blastP with an e-value cutoff of $1e^{-10}$ and minimal identity of 50%. Reference sequences included thiosulfate reductase PhsA from *Salmonella typhimurium* [39], thiosulfate reductase from *Nitratidesulfovibrio vulgaris* strain *Miyazaki F* and strain *Hildenborough* [40], Sox multienzyme system and thiosulfate dehydrogenase TsdA from *Paracoccus thiocyanatus* SST [41], Hdr-like enzyme from *Hyphomicrobium denitrificans* [42], rhodanese-like sulfurtransferase SbdP from *Aquifex aeolicus* [43], thiosulfohydrolase SoxB, and sulfur dioxygenase SdoAB homologs from *Erythrobacter flavus* 21-3 [44].

Proteomics

Harvested cells were disrupted using a UP50H – Compact Lab Homogenizer (Hielscher Ultrasound Technology, Germany) at cycle 0.5 and amplitude of 100%. Cell debris was removed by centrifugation (10 min, 10,000 x *g*) and the crude cell extracts were submitted to total proteomic analysis at the Proteomics Centre of the University of Konstanz as described previously [45]. The samples were analyzed on an Orbitrap Fusion with EASY-nLC 1200 (Thermo Fisher Scientific), and Tandem mass spectra were searched against the proteins inferred from the LT0009 genome using Mascot (Matrix Science) and Proteome Discoverer v 1.3

(Thermo Fisher Scientific) with Trypsin enzyme cleavage, static cysteine alkylation by chloroacetamide, and variable methionine oxidation.

Transcriptomics

RNA from triplicate LT0009 cultures grown with 10 mmol/l of taurine, sulfolactate, or thiosulfate was extracted using the Analytic Jena innuprep RNA Mini Kit 2.0 following manufacturer's instructions (JMF project JMF-2012-1). Extracted RNA was subjected to Turbo DNAse (Ambion) treatment to remove residual DNA contamination. Ribosomal RNA was not depleted to minimize sample processing biases. Stranded total RNA libraries were prepared using the NEBNext Ultra II Directional RNA Library Prep Kit for Illumina following the manufacturer's instructions. Paired-end (150 cycles) sequencing was performed on the HiSeq 3000 (Illumina). Raw reads were quality-filtered by removing adaptor-contaminated and low-quality reads at a Phred score of 28 using the bbduk function of BBMap (version 37.61) [46]. Next, filtered sequences were mapped as paired reads with a minimal identity of 99% to a reference file of all open reading frames of the LT0009 genome using the bbmap function of BBMap. Normalized expression levels of transcripts were depicted as transcripts per million (TPM) values [47]. Read count values were used as input data for differential expression analysis by DESeq2 [48].

***Taurinivorans muris*- and *Bilophila wadsworthia*-related sequences in 16S rRNA gene amplicon datasets of human and animal guts**

Occurrence and prevalence of 16S rRNA gene sequences related to LT0009 and *B. wadsworthia* ATCC 49260 were analyzed with the Integrated Microbial Next-Generation Sequencing (IMNGS) platform [49]. All Sequence Read Archive (SRA) amplicon sequence datasets containing the word "gut" in the "Origin" field were used for further analyses. A 97% sequence similarity cut-off was used to identify related 16S rRNA gene sequences in the "gut" dataset that contained approximately 5.3 billion sequences from 123,723 gut samples, including 81,501 gut samples with host information. Further information on mouse studies with at least 20 samples that were positive for *B. wadsworthia* was manually compiled from the NCBI SRA entries or the corresponding publications (Supplementary Data 8).

LT0009-centric gut metatranscriptome analyses of laboratory mice

Cecal and fecal metatranscriptomes from a high-glucose diet experiment in mice (HG study) (JMF project JMF-2101-5) were analyzed for LT0009 gene expression. Mouse experiments were conducted following protocols approved by the Austrian government (BMWF-66.006/ 0032-WF/V/3b/2014). Six female C57BL/6 mice (10-11 weeks old) were randomly allocated to groups and fed either a control diet (5% glucose; n = 3) or a high-glucose diet (65% glucose; n = 3) for about 3 weeks. All mice were housed in the Max Perutz

Laboratories, University of Vienna, in a controlled environment (14 /10 h day/night cycle, 21 °C and 50-60% humidity). Cecal and fecal contents were collected for RNA extraction. RNA was extracted as previously described [50]. Ribosomal RNA was depleted using the RiboZero rRNA depletion Kit (Illumina), and stranded RNA libraries were prepared using the NEBNext Ultra II Directional RNA Library Prep Kit for Illumina following manufacturers' instructions, and sequenced on an Illumina HiSeq 3000 in paired-end mode (75 + 91 bp). Library preparation, sequencing, and data processing were performed at the Joint Microbiome Facility of the Medical University of Vienna and the University of Vienna.

We additionally re-analyzed mouse gut metatranscriptomes from a previous study for LT0009 gene expression (Plin2 study) [51]. Sequence data (PRJNA379425) derived from eight-week-old C57BL/6 wild-type and Perilipin2-null (Plin2) mice fed high-fat/low-carbohydrate or low-fat/high-carbohydrate diets. Low-quality reads were removed at a Phred score of 28 using the bbduk function of BBMap (version 37.61) and filtered sequences were mapped to the LT0009 genome using the bbmap function of BBMap with a 99% similarity cutoff [46]. Expression levels of transcripts were normalized as TPM values for comparison.

Re-analysis of metagenome and 16S rRNA gene amplicon data from Stacy *et al.* 2021

Gut metagenomics data (NCBI BioProject accessions PRJNA390686 and PRJNA666931) of mouse models that were previously shown to enhance H_2S-mediated resistance to *Klebsiella pneumoniae* and/or *Citrobacter rodentium* [52,53] were re-analyzed with regards to the identity and relative abundance of *dsrAB*-encoding bacteria. Briefly, the metagenome reads were mapped to customized *dsrA* and *dsrB* gene databases, which comprised species-level gene sequences from genomes of the Genome Taxonomy Database (GTDB release R95) [54] and from *T. muris* LT0009*,* using bbmap (v. 39.01) [55]. The read counts of each species-level reference gene were used to calculate the relative abundance of *dsrA*- and *dsrB*-containing taxa in each metagenome.

We further reanalyzed 16S rRNA gene amplicon sequencing data of the second (PRJNA390686) [53] and the tenth generation of wildR mice (PRJNA666931) [52]. Downloaded fastq files were run through the DADA2 pipeline [56]. The sequences were filtered with parameters maxN = 0, maxEE = c (2,2), truncQ = 10, and truncLen = c (180, 150). Following chimera removal, taxonomy was assigned to the amplicon sequence variants (ASVs) using the silva_nr99_v138 reference database [13]. ASVs assigned to unclassified *Desulfovibrionaceae* spp. were blasted against the 16S rRNA gene sequence of strain LT0009. ASVs with a similarity of >98% to the LT0009 16S rRNA gene sequence were re-classified as *T. muris*.

Gnotobiotic Oligo-Mouse-Microbiota mouse experiments

Twelve gnotobiotic C57BL/6 mice (7-13 weeks old) were used for the experiment, which was approved by the local authorities (Regierung von Oberbayern; ROB-55.2-2532.Vet_02-20-84). Mice were housed under

germ-free conditions in flexible film isolators (North Kent Plastic Cages) or in Han-gnotocages (ZOONLAB) at a twelve hour light-dark cycle at 22 ± 1.5°C and 50 ± 5 % humidity. The mice were supplied with autoclaved ddH_2O and Mouse-Breeding complete feed for mice (Ssniff) *ad libitum*. Strain LT0009 was cultivated in Anaerobic Akkermansia Medium [57] supplemented with 10 mmol/l taurine, 20 mmol/l sodium pyruvate, and 200 µg/l naphthoquinone. A subculture was incubated at 37°C for 3 days. Mice (n = 6, 3 female and 3 male) stably colonized with OMM[12] strains were orally (50 µl) and rectally (100 µl) inoculated with the LT0009 subculture and the control group (n = 6, 4 female and 2 male) was treated with the same volume of sterile 1x phosphate-buffered saline. After 10 days, the mice were infected with the human enteric pathogen *Salmonella enterica* serovar Typhimurium (avirulent *S. enterica* Tm strain M2702; 5×10^7 c.f.u.). At the same time point, the fecal microbiota composition was determined by strain-specific qPCR assays as previously described [58]. DNA was extracted using a phenol-chloroform based protocol as described previously [59].

New 16S rRNA gene-targeted primers (forward: 5'-TTCGGATCGTAAACCTCTGTCA-3'; reverse: 5'-GGTACCGTCAATTCACTGCTGAT-3') and a detector probe (5' 6-carboxyhexafluorescein-CAGGGAAGAACGGTCAC-black hole quencher 1-3') for qPCR of LT0009 were designed using Primer Express 3 (Applied Biosystems, Life Technologies). The qPCR probe/primers for LT0009 were validated as described previously [58]. Briefly, qPCR conditions were established according to the MIQE guidelines [60]. All primers were designed for an optimal annealing temperature of 60°C. Standard curves were determined using linearized plasmid as DNA template. Plasmid DNA was diluted in H_2O containing 100 µg/µl yeast t-RNA (Roche). Standard curves were determined on a Roche Lightcycler96 instrument in triplicates. Efficiency of each qPCR reaction was calculated based on the slope of standard curves (qPCR efficiency: $(10^{(-1/\text{slope of standard curve})}-1) \times 100$) using 10-fold dilutions of template. Efficiencies for all qPCR reactions were within the range of 90-110 %. For all experiments, the software Lightcycler96 version 1.1. reproduced standard curves based on single DNA template with known DNA quantity as well as the efficiency derived from the standard curve of each qPCR assay of the initial run of the standard curves. Specificity was confirmed by performing an assay for each primer/probe pair using an equimolar mixture of all linearized plasmids except for the one to be tested as a template. One PCR reaction (total volume: 20 µl) contained 5 ng DNA as template, 300 µM of each primer, and 250 µM of the corresponding hydrolysis probe.

The mice were sacrificed by cervical dislocation two days post infection (p. i.). Abundance of viable *S. enterica* Tm at 24 and 48 h p.i. in the feces and at 48 h p.i. in the cecal content was determined by plating [58]. Fecal samples of three mice from each group form day two p.i. were used for metatranscriptome sequencing (JMF project JMF-2104-01) and fecal samples from the other three mice from each group were used for taurine and bile acids quantification (see below). RNA was extracted using the Analytic Jena innuprep RNA Mini Kit 2.0 following manufacturer's instructions. Extracted RNA was subjected to Turbo

DNAse (Ambion) treatment to remove residual DNA contamination. Ribosomal RNA was depleted using the RiboZero rRNA depletion Kit (Illumina), and stranded RNA libraries were prepared using the NEBNext Ultra II Directional RNA Library Prep Kit for Illumina following manufacturers' instructions, and sequenced on an Illumina Novaseq 6000 in paired-end mode (2 x 100 bp). Reads were filtered for contamination and adapters using BBDuk as follows: k = 23, mink = 11 (https://sourceforge.net/projects/bbmap/). Reads after quality control were mapped to the reference genomes of individual strains [61] and *S. enterica* Tm (GCA_000210855.2) using BBMap (version 38.92) [46] at 98% sequence identity. Genes of OMM[12] strains and *S. enterica* Tm that were significantly differentially expressed between mice with or without strain LT0009 were revealed using Deseq2 (version 1.36.0) and plotted using R (version 4.2.1)[48]. Sequences of eight representative bile salt hydrolase (BSH) genes from the human gut microbiome [62] were used to produce the BLASTP search database and used to identify BSH homologs with >30% identity in genomes of the gnotobiotic community (Supplementary Data 9). Prophage regions in *E. clostridioformis* YL32 were predicted using PHASTER [63].

Mono- and co-colonization experiments in germ-free mice

Animal experiments were approved by the local authorities (Regierung von Oberbayern; ROB-55.2-2532.Vet_02-20-84). Mice were housed under germ-free conditions in flexible film isolators (North Kent Plastic Cages) or in Han-gnotocages (ZOONLAB). The mice were supplied with autoclaved ddH_2O and Mouse-Breeding complete feed for mice (Ssniff) ad libitum. For *T. muris* LT0009 mono- and co-colonization experiments, seven female mice of 10-20 weeks of age were used and animals were randomly assigned to experimental groups. Mice were kept in groups of three and four mice/cage during the experiment. All animals were scored twice daily for their health status. Mice were mono-associated with LT0009 (n = 4) or co-colonized with LT0009, *Bacteroides caecimuris* I48, and *Enterococcus faecalis* KB1 (n = 3) and were inoculated two times (72 h apart) with the bacterial mixtures (OD 600nm adjusted to a 1:1:1 ratio) by gavage (50 µl orally, 100 µl rectally). Fecal samples were collected for strain-specific qPCR on days 3 and 7. All mice were sacrificed 10 days after initial colonization. Intestinal content from ileum, cecum, colon and feces was harvested, weighed and frozen at -20°C before DNA extraction for strain specific qPCR. Further, intestinal content from ileum, cecum, colon and feces was weighed, snap-frozen in liquid nitrogen and stored at -80°C for taurine and bile acids quantification (see below). The absolute abundance of the inoculated strains was determined by strain-specific qPCR assays as described above.

Bile acids and taurine quantification in mouse gut content

Gut content and fecal samples were manually homogenized using a spatula and dried at 4°C with a SpeedVac concentrator (Labconco), followed by the addition of 65 µL of methanol/acetonitrile/H_2O

(40:40:20; v:v:v) for each mg of dried sample. The extraction consisted of 20 s in a bead shaker (FastPrep-24 5G, 6 m/s) followed by sonication in an ice bath for 10 min and storage at -20 C° overnight. Afterwards the samples were centrifuged at 18,000 g and 4°C for 10 min and transferred to a new tube. The remaining solution was evaporated to dryness at 4°C in a SpeedVac (Labconco) and the residues were reconstituted with (ACN/H_2O, 50:50, v:v), containing an internal standard ($^2H_4$-ursudeoxycholic acid, 2 µM), to the same volume before evaporation, vortexed and sonicated in an ice bath for 15 min. After centrifugation at 18,000 g and 4°C for 10 min, the samples were transferred to HPLC vials and stored at -80°C until analysis. For the targeted LC-MS/MS analysis a Dionex Ultimate 3000 UHPLC (Thermo) system coupled to a TSQ Vantage triple quadrupole mass spectrometer (Thermo) in negative ionization mode via an electrospray ionization interface was applied. The method was based on the assay established by Pristner et al. [64]. An Atlantis T3 column (Waters, 3 µm, 3 mm × 150 mm) at a flow rate of 0.6 ml/min, and a column oven temperature of 40°C. The injection volume was 5 µL. Chromatography used H_2O with 0.1% formic acid (eluent A) and ACN/MeOH (1:1, v:v, eluent B) with 2% H_2O and 0.1% formic acid for the following gradient: 0 - 1 min at 5% B; rise to 50% B until 1.5 min; increase to 95% B until 10 min; 10 - 13 min at 95% B; 13 - 13.1 min from 95% B to 75% B and maintain until 19 min before re-equilibrating at 5% B until 21 min. The following ion source parameters were used: Capillary temperature of 300 (°C), vaporizer temperature of 300 (°C), sheath gas pressure of 40 (Arb), aux valve flow of 10 (Arb), declustering potential of 14 (V), collision gas pressure of 1.5 (mTorr) and a spray voltage of 3000 (V). The cycle time was set to 0.6 (s), and Q1 and Q3 peak width was kept at 0.7 (FWHM). Each sample was measured in triplicate and the raw files obtained from the LC-MS/MS experiments were processed and quantified using the software Skyline [65]. The raw data is publicly available under https://phaidra.univie.ac.at/o:1649944. The absolute concentrations of taurine and selected bile acids were calculated employing a dilution series of matrix samples spiked with reference standards comprising deoxycholic acid (Sigma-Aldrich, cas.no. 83-44-3), ursodeoxycholic acid (Sigma-Aldrich, cas.no. 128-13-2), lithocholic acid (Sigma-Aldrich, cas.no. 434-12-9), chenodeoxycholic acid (Sigma-Aldrich, cas.no. 474-25-9), hyodeoxycholic acid (Sigma-Aldrich, cas.no. 83-49-8), and cholic acid (Sigma-Aldrich, cas.no. 81-25-4).

Growth experiment of *Enterocloster clostridioformis*

Enterocloster clostridioformis YL32 was grown anaerobically (85% N_2, 10% CO_2, 5% H_2) in brain-heart-infusion medium at 37°C. Growth was monitored by spectrophotometric measurement of the OD_{600nm}. In a preliminary experiment, mitomycin C was added to the culture during the onset of the exponential phase. Subsequent growth was tracked in order to examine the suitability of mitomycin C as a positive control for prophage induction. In the main experiment, sodium sulfide (Na_2S) was added to a final concentration of 0.5 mM or 5 mM to growing cultures at an OD_{600nm} of 0.15-0.25. Control tubes remained unamended. All

treatments were performed in triplicates. Samples from the cultures were frozen immediately in liquid nitrogen for subsequent quantification by droplet digital PCR (ddPCR). Sulfide concentrations were quantified at 30 h and 124 h. DNA was extracted from 0.2 ml of culture using the DNeasy blood & tissue kit (Qiagen) according to the manufacturer's instructions and quantified using the Qubit dsDNA BR Assay kit and the Qubit 4 Fluorometer (Invitrogen). Primers targeting two host genes (*era*, *recA*) and two genes of the prophage Saumur (*cysH*, *clpP*) were designed and their annealing temperatures and input DNA amount were optimized by gradient PCR (Supplementary Data 9). The specificity of each ddPCR assay was validated through isolation of the ddPCR product using liquid nitrogen as described previously [66] and subsequent Sanger sequencing. One PCR reaction (total volume of 22 µl) contained 0.1 ng of extracted DNA, 11 µl 2x EvaGreen supermix (BioRad), 0.2 µl of each primer (10 µM), and 8.6 µl nuclease-free water. PCR consisted of an initial step at 95°C for 5 min, followed by 40 cycles of denaturation at 95°C for 30 s and annealing/elongation at the primer-specific temperature for 1 min, and a final step for signal stabilization at 4°C for 5 min and at 90°C for 5 min. The ramp rate was set to 2 °C per second for all steps. Droplets were created using the QX200 Droplet generator (BioRad). The Qx200 Droplet reader (Biorad) was used for ddPCR read out. At least 12,000 accepted droplets per sample were required for a successful run. Gene abundance was normalized using the respective DNA extraction yield and depicted as copy number per ml of culture.

Additional energy metabolism of *Taurinivorans muris* LT0009

Genome reconstruction of LT0009 suggested the potential to utilize lactate, pyruvate, and H_2. We experimentally confirmed that lactate and pyruvate are used as electron donors for taurine respiration (Fig. 2b). LT0009 expressed lactate permease LutP and L-lactate dehydrogenase LutABC/LldEFG for lactate import and oxidation to pyruvate (Fig. 2a, Supplementary Data 1) [67,68]. A homolog of another putative lactate dehydrogenase, the flavin and iron–sulfur containing membrane-associated oxidoreductase Dld-II [69] is also encoded by LT0009 (TAU_v1_1652), yet was not expressed (Supplementary Data 1). Pyruvate can be further oxidized by LT0009 to acetyl-CoA with either pyruvate:ferredoxin oxidoreductase Por or pyruvate formate-lyase PflD. Growth experiments of LT0009 with or without pyruvate revealed that additional pyruvate can stimulate its growth (Supplementary Fig. 3b).

For comparison, *B. wadsworthia* strains can grow with lactate, pyruvate, formate, and H_2 as electron donors [3,45,70]. Strain LT0009 also used formate as electron donor for taurine-respiration (Fig. 2b), yet its formate metabolism remains unresolved as genes for formate dehydrogenase or the formate hydrogen-lyase complex were not detected. LT0009 encodes four hydrogenases (Supplementary Data 3). The presence of genes for a respiratory, H_2-uptake group 1b [NiFe]-hydrogenase (*hybAC*) and its corresponding maturation factor *hypABCDEF* is consistent with the prevalence of this enzyme in hydrogenotrophic *Desulfobacterota* in the gut [71] and suggested H_2 as an additional electron donor for LT0009. However, strain LT0009 did not grow with H_2 as electron donor under the conditions we used (Fig. 2b). Besides the potential for H_2-utilization, LT0009 also encodes a fermentative group A1 [FeFe] hydrogenase and a group C3 [FeFe] hydrogenase of yet undetermined biochemical function [19]. The presence of *cooMKLXUHF* operon encoding H_2-evolving group 4c [NiFe] carbon monoxide-induced hydrogenase suggested carbon monoxide, a ubiquitous molecule in the gut [72], as a potential electron donor and source of H_2 for LT0009. However, LT0009 lacks the gene cluster *cooFSCTJ* that encodes carbon monoxide dehydrogenase CooS and the electron-transfer protein CooF, which is required for optimal H_2 production by the carbon monoxide-induced hydrogenase in *Rhodospirillum rubrum* [73,74].

LT0009 encodes a complete glycolysis/gluconeogenesis pathway. The coupling of electron transfer to energy conservation is likely mediated by an H^+/Na^+-pumping Rnf complex (RnfCDGEAB) [75] and ATP synthase (AtpABCDEFGH).

Colonization of *Taurinivorans muris* LT0009 in the mouse gut depends on deconjugation of taurine-conjugated bile acids by other community members

Taurine liberated from bile acids by BSH-expressing bacteria is a main source of taurine in the gut [87]. We performed *in vitro* growth experiments with OMM^{12} spent media and *in vivo* colonization experiments with germ-free mice to investigate the metabolic dependency of *T. muris* LT0009 on other gut community members. Consistent with the absence of BSH genes in its genome, strain LT0009 did not grow with TCA as substrate in the medium (Supplementary Fig. 7a). In contrast, spent medium that has been supplemented with TCA prior to growth of OMM^{12} strains contained free taurine and supported growth and H_2S production of LT0009 (Supplementary Fig. 7b and c). This re-confirmed the capability of the OMM^{12} strain consortium for deconjugation of taurine-conjugated bile acids [88] and further indicated that taurine-based *T. muris* ecophysiology in the gut is dependent on taurine-releasing bacteria.

Next, we performed colonization experiments in germ-free mice with either strain LT0009 alone or with strain LT0009 and the two taurine-conjugated-bile-acids-deconjugating OMM^{12} strains *Bacteroides caecimuris* I48 and *Enterococcus faecalis* KB1 (Supplementary Fig. 8a). Although free taurine was detected in the ileum (5.4 ± 5.2 µmol/g, mean ± SD), cecum (0.8 ± 0.2 µmol/g), and colon (2.8 ± 2.3 µmol/g), strain LT0009 alone did not colonize the germ-free mice, which thus essentially remained germ-free throughout the experiment (Supplementary Fig. 8b and c). Free taurine occurs in tissues of germ-free rodents and derives mostly from the host and less from the diet [89–91]. Deconjugated bile acids were not detected or very low abundant in the mono-associated mice, which supports previous findings from germ-free rodents that microbiota absence shifts the bile acid profile to dominance of taurine-conjugated species such as TCA [90–92]. In stark contrast to mono-associated mice, the strain mixture of LT0009, I48, and KB1 successfully co-colonized germ-free mice during the ten days of the experiment. LT0009 reached a stable population abundance later (day 7) than strains I48 and KB1 (day 3). Final abundances (day 10) of all strains increased gradually from ileum to feces (Supplementary Fig. 8b). Gut samples of co-colonized mice contained significantly more deconjugated and less taurine-conjugated bile acids than gut samples of mono-associated mice, while no significant differences in taurine concentrations between mono-associated and co-colonized mice were observed (Supplementary Fig. 8c). This differential taurine and bile acids abundance pattern suggests that significant amounts of free taurine were (i) produced from taurine-conjugated bile acids and (ii) also largely consumed in the intestinal tract of co-colonized mice.

Together, our results show that successful intestinal colonization of *T. muris* LT0009 in germ-free mice is strictly dependent on other gut bacteria. The LT0009 growth-enabling effect of these bacteria relies on producing sufficient amounts of free taurine from bile acid deconjugation, but potentially also on providing generally favorable growth conditions for this fastidious anaerobe, such as reduced oxygen concentrations in the gut.

Sulfide impacted growth of *Enterocloster clostridioformis* YL32 in pure culture but did not activate its prophage Saumur

The colonization of *T. muris* LT0009 in gnotobiotic OMM[12] mice led to increased transcription of the *E. clostridioformis* YL32 prophage Saumur (Figure 4), which is part of the temporally stable viral community in the OMM[12] consortium [76,77]. Addition of sulfide to a *Lactococcus lactis* culture was previously shown to increase production of viable particles of phage P087 [78]. We thus (i) speculated that sulfide could be a yet unrecognized inducer of prophage activity [79] and (ii) tested in growth experiments if sulfide could induce the prophage Saumur in the strain *E. clostridioformis* YL32. Initial growth tests of *E. clostridioformis* were performed with mitomycin C, which is a commonly used prophage inducer [80]. However, addition of mitomycin C at concentrations from 0.25 µg/ml to 1 µg/ml did not have a growth-inhibiting effect on *E. clostridioformis* compared to an unamended control culture and could thus not be used as a positive control (data not shown). Subsequently, we tested the impact of sulfide at two concentrations (0.5 mM, 5 mM), which are in the range of physiological sulfide concentrations in the gut [81–85] (Supplementary Fig. 10a). Sulfide concentrations in the amended cultures did not change between 30 h and 124 h of incubation, which indicated that sulfide was not lost from the culture (Supplementary Fig. 10b). Notably, small amounts of H_2S (maximally 0.28 mM) were produced by *E. clostridioformis* YL32 in the control, potentially from degradation of cysteine as suggested previously [86]. Cultures amended with 5 mM Na_2S showed decreased growth compared to the unamended controls. In contrast, cultures amended with 0.5 mM Na_2S surprisingly exhibited a higher maximal OD_{600nm} and maintained a higher OD_{600nm} throughout the stationary phase compared to the unamended controls (Supplementary Fig. 10a). However, quantification of *E. clostridioformis* YL32 by ddPCR did not confirm this growth curve pattern, but showed that only 5 mM Na_2S had a significant impact on growth (Supplementary Fig. 10c). Growth at 0.5 mM Na_2S was not different from the control. This suggested that optical density measurements are an inaccurate measure of growth of this strain [83–85]. The growth inhibition occurred immediately after addition of 5 mM Na_2S and thus differed from the delayed response commonly observed upon prophage induction [82,86–88]. The abundance profiles of two host genes and two prophage Saumur genes of strain YL32 were very similar for each treatment (Supplementary Fig. 10c). Additionally, the prophage-to-host gene ratio did not deviate notably from a value of one (Supplementary Fig. 10d), which suggested that the prophage Saumur was inactive across all treatments. The growth-reducing impact of 5 mM Na_2S on *E. clostridioformis* YL32 was thus most likely attributed to the dose-dependent toxic effects of sulfide [86].

Description of *Taurinivorans* gen. nov.

Taurinivorans gen. nov. (Tau.ri.ni.vo'rans. N.L. n. *taurinum*, taurine; L. part. adj. *vorans*, eating; N.L. masc. n. *Taurinivorans*, a taurine eater). Comparative genome analyses suggest the common electron acceptor is taurine, which is degraded and reduced to sulfide via the Tpa-Xsc-DsrAB-DsrC pathway. Type species: *Taurinivorans muris* sp. nov., family: *Desulfovibrionaceae* VP, order: *Desulfovibrionales* VP (T) emend., class: *Desulfovibrionia* class. nov., phylum: *Desulfobacterota* phyl. nov. [93].

Description of *Taurinivorans muris* sp. nov.

Taurinivorans muris sp. nov. (mu'ris. L. gen. n. *muris*, of a mouse, referring to its origin from the mouse intestine). The type strain is strain LT0009^T (= DSM 111569 = JCM 34262), isolated from the mouse gut with taurine as the electron acceptor and lactate/pyruvate as electron donors. Formate was also used as an electron donor for taurine respiration. Cells are Gram-stain-negative, spirilloid in shape, and motile by means of lophotrichous polar flagella. The temperature range is 27-42°C and the optimum pH is 6.5 (range 6-8.5) for strictly anaerobic growth. The optimal taurine concentration for growth is 40 mmol/l, higher taurine concentrations inhibit growth. Sulfolactate and thiosulfate are additional electron acceptors for anaerobic respiration and are also reduced to hydrogen sulfide. Yeast extract and 1,4-naphthoquinone are required as growth supplements for laboratory cultivation of the isolate. Its genome size is 2.2 Mbp with a G+C content of 43.6%. The GenBank accession numbers for the genome and the 16S rRNA gene sequence of strain LT0009^T are CP065938 and MW258658, respectively.

Supplementary Figure S1

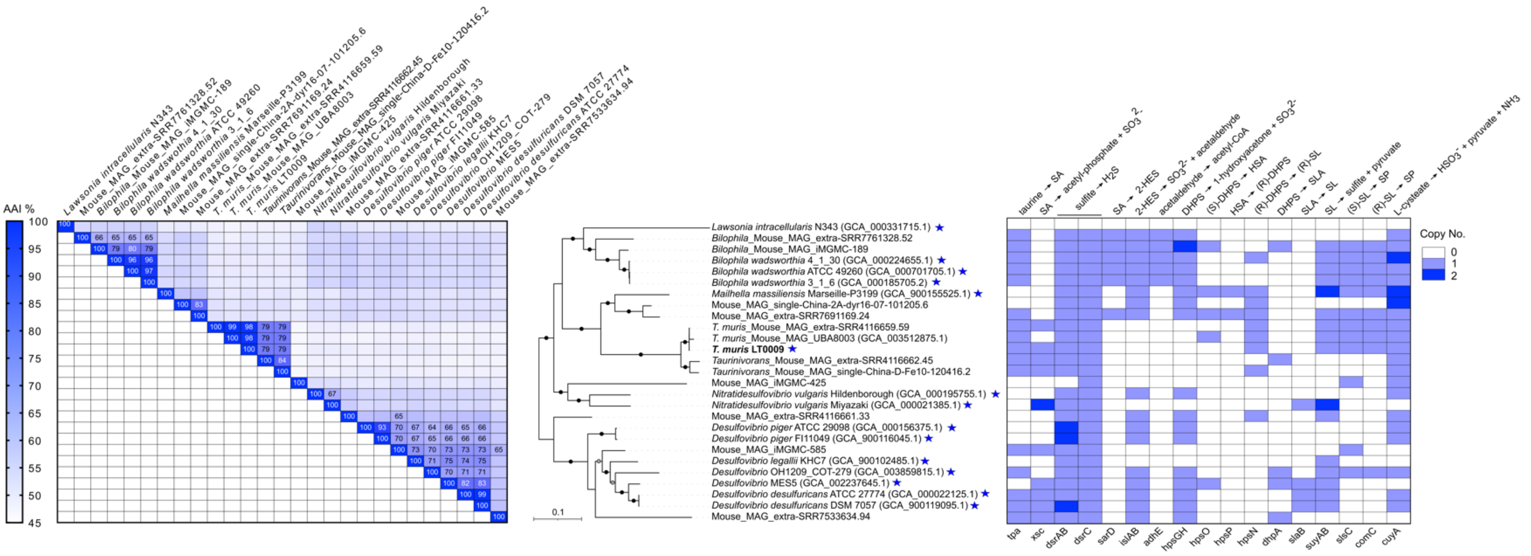

Supplementary Figure 1. Average amino acid identities, phylogenomic relationship, and organosulfonate metabolism gene distribution of strain LT0009 and related genomes/MAGs of the *Desulfovibrio-Mailhella-Taurinivorans-Bilophila* lineage. Left panel. Matrix of pairwise AAIs; only values ≥63.4 (genus cut-off [81]) are shown. Middle panel. Phylogenomic tree from Figure 1b is used here to illustrate the AAI and sulfur metabolism gene similarity of the close relatives. Right panel. Presence/absence of organosulfonates metabolism genes in the genomes of LT0009 and its relatives. Organosulfonate metabolism genes and encoded enzymes/proteins: *tpa*, taurine:pyruvate aminotransferase; *xsc*, sulfoacetaldehyde acetyltransferase; *dsrAB*, dissimilatory sulfite reductase subunits A and B; *dsrC*, sulfite reduction co-substrate DsrC; *sarD*, sulfoacetaldehyde reductase; *islAB*, isethionate sulfite-lyase complex; *ahdE*, CoA-acylating aldehyde dehydrogenase; *hpsGH*, DHPS sulfite-lyase complex; *hspNOP*, DHPS dehydrogenases; *dhpA*, NAD$^+$-dependent DHPS dehydrogenase; *slaB*, 3-sulfolactaldehyde dehydrogenase; *suyAB*, (R)-sulfolactate sulfo-lyase; slsC, (S)-sulfolactate dehydrogenase; comC, (2R)-3-sulfolactate dehydrogenase; *cuyA*, L-cysteate sulfo-lyase. SA, sulfoacetaldehyde; 2-HES, 2-hydroxyethane-1-sulfonic acid (isethionate); DHPS, 2,3-dihydroxypropane-1-sulfonate; HSA, 2-oxo-3-hydroxy-propane-1-sulfonate; SL, 3-sulfolactate; SLA, 3-sulfolactaldehyde; SP, sulfopyruvate.

Supplementary Figure S2

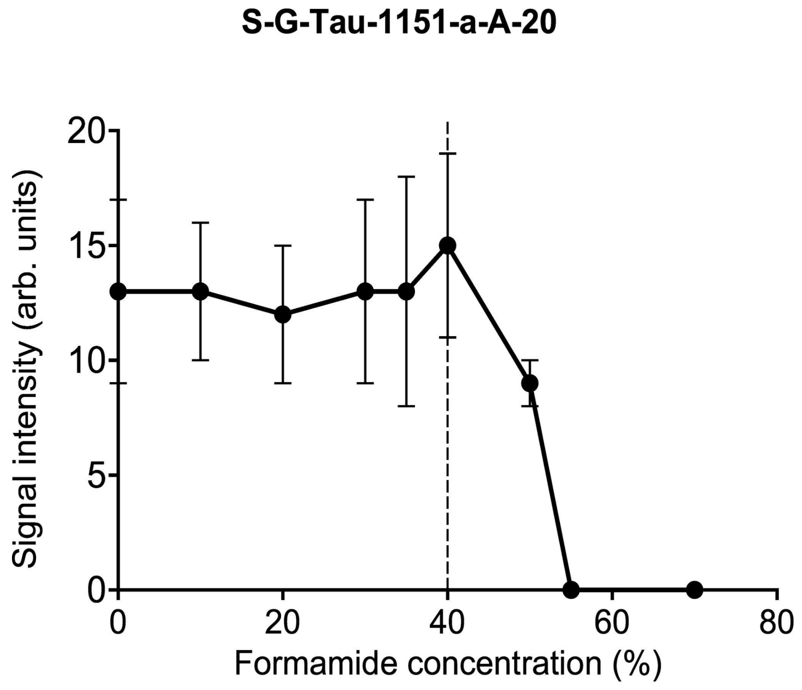

Supplementary Figure 2. Formamide dissociation profile of FISH probe TAU1151. Fluorescence signal intensities (arb. units, arbitrary units) under increasing formamide concentrations are depicted for strain LT0009 hybridized with the Cy3-labeled probe S-G-Tau-1151-a-A-20 (TAU1151) for the genus *Taurinivorans*. The dashed vertical line indicates the formamide concentration for the best possible specificity and sensitivity of probe TAU1151. Data are presented as mean ± standard deviation. For each formamide concentration, signal intensities were calculated from individual cells of recorded images of at least five randomly chosen fields of view (0%, n = 697 cells; 10%, n = 394 cells; 20%, n = 394 cells; 30%, n = 145 cells; 35%, n = 145 cells; 40%, n = 145 cells; 50%, n = 15 cells; 55%, n = 15 cells; 70%, n = 15 cells). Source data are provided as a Source Data file.

Supplementary Figure S3

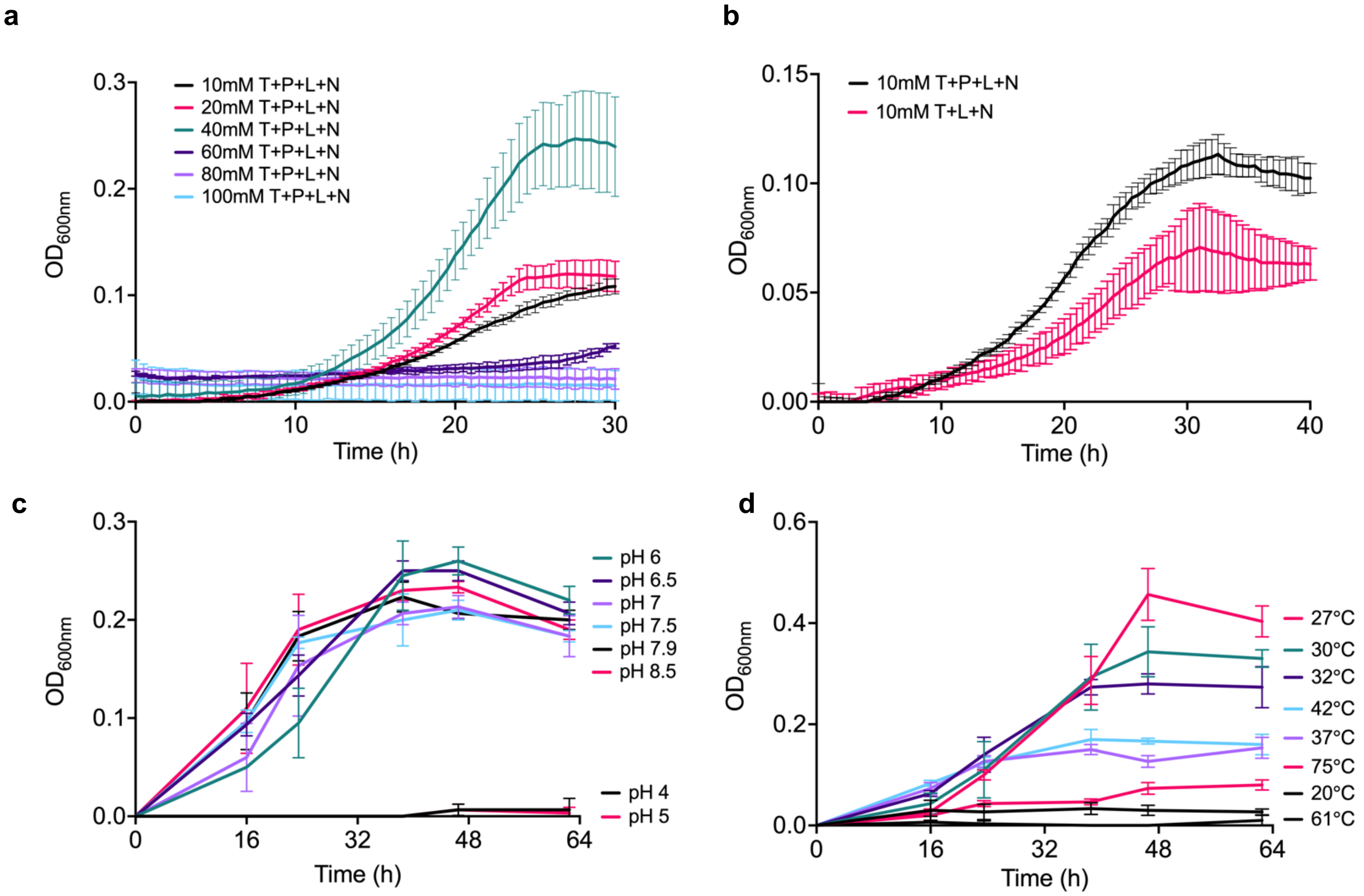

Supplementary Figure 3. Anaerobic growth tests of *Taurinivorans muris* LT0009. LT0009 grown **a.** with different taurine concentrations (10, 20, 40, 60, 80, and 100 mmol/l) and 20 mmol/l pyruvate, 10 mmol/l lactate, and 0.2 mg/l 1,4-naphthoquinone **b.** with or without pyruvate **c.** at different pH values (temperature 37°C) and **d.** at different temperatures (pH 7.2). Growth curves show the averages of optical density measurements at 600 nm (OD_{600nm}) in triplicate culture. Error bars represent one standard deviation. T, taurine; P, pyruvate; L, lactate; N, 1,4-naphthoquinone. Source data are provided as a Source Data file.

Supplementary Figure S4

a

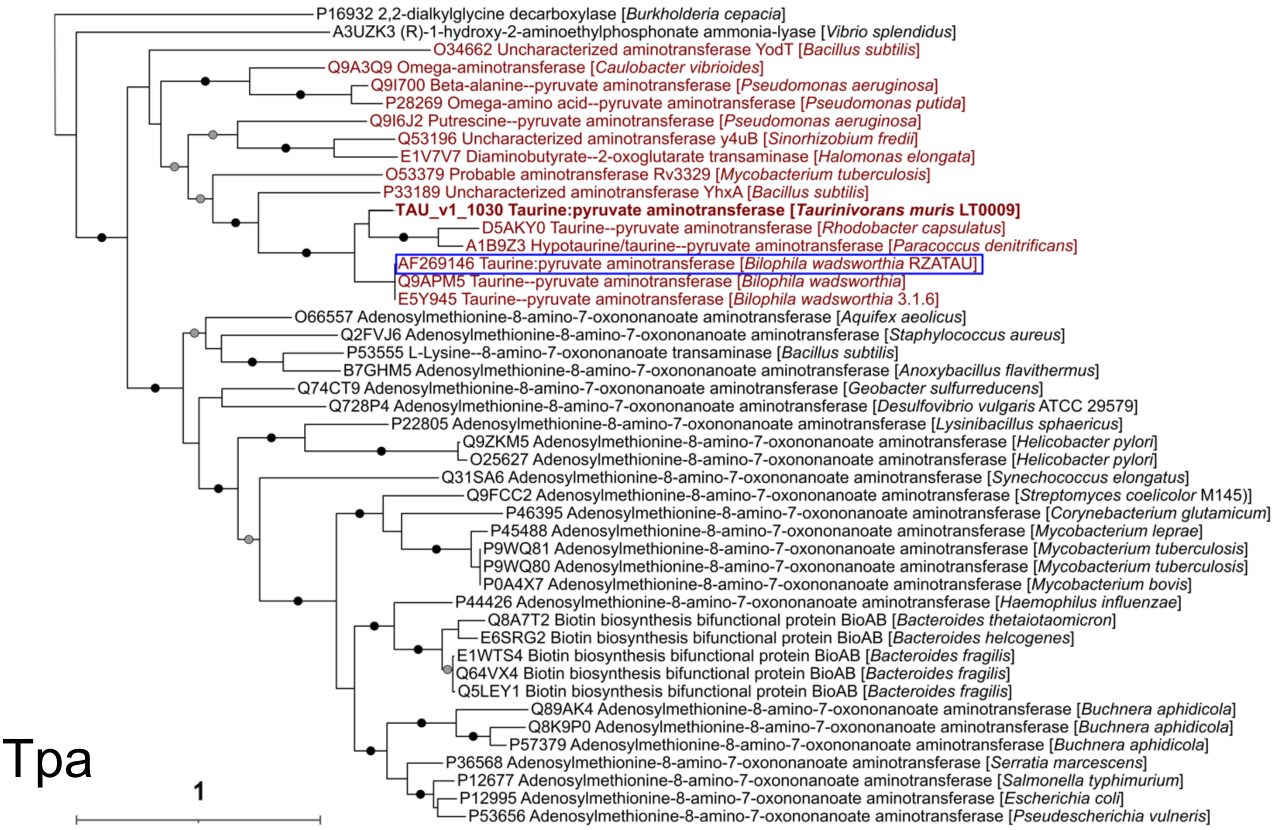

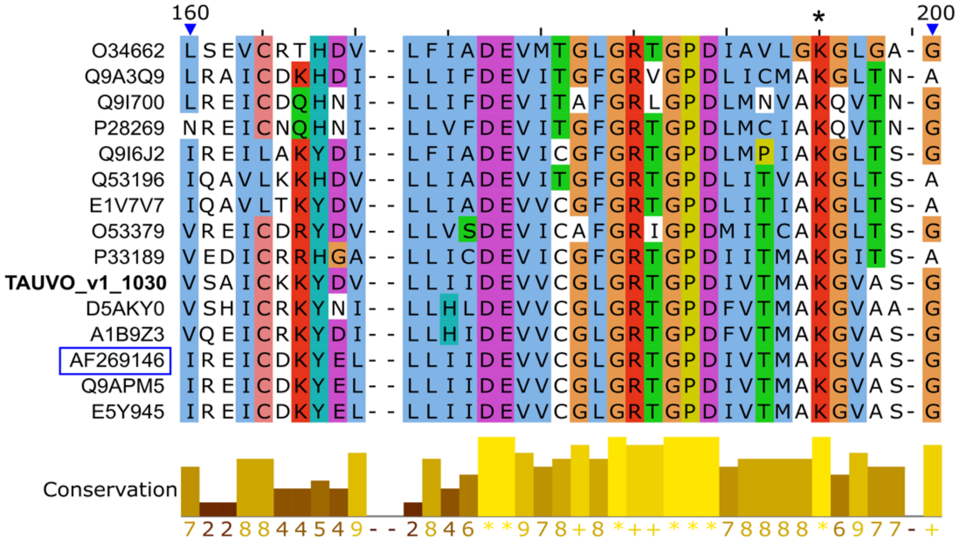

Supplementary Figure S4

b

Xsc

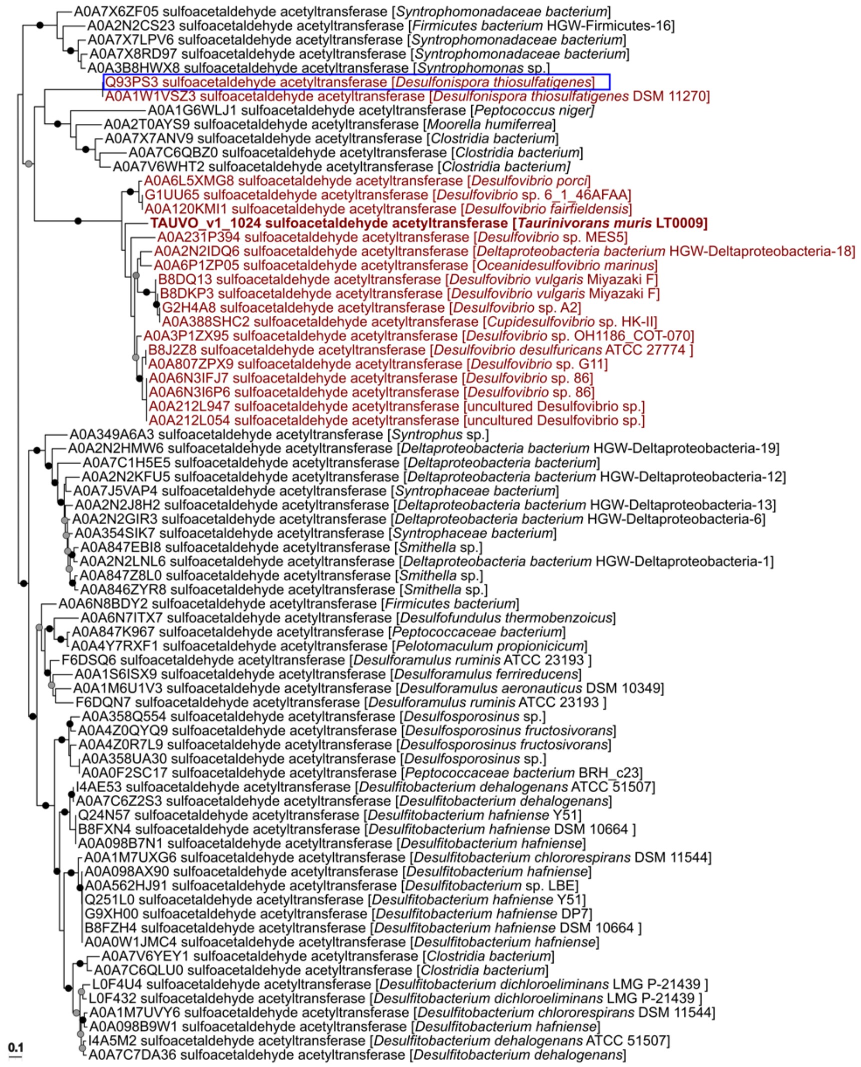

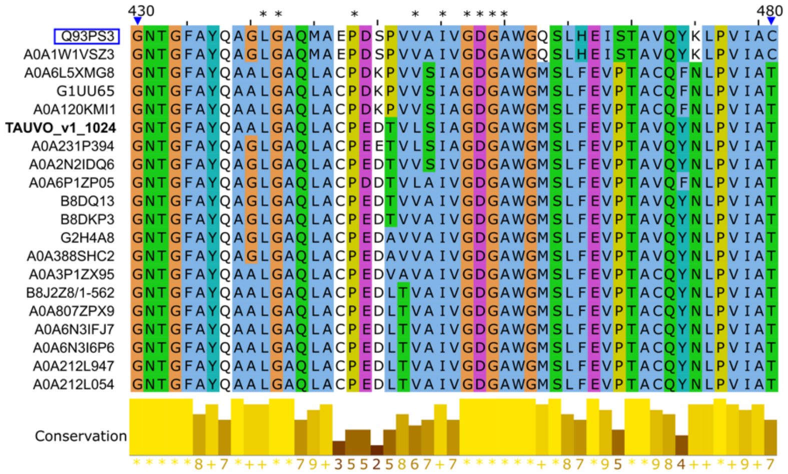

Supplementary Figure S4

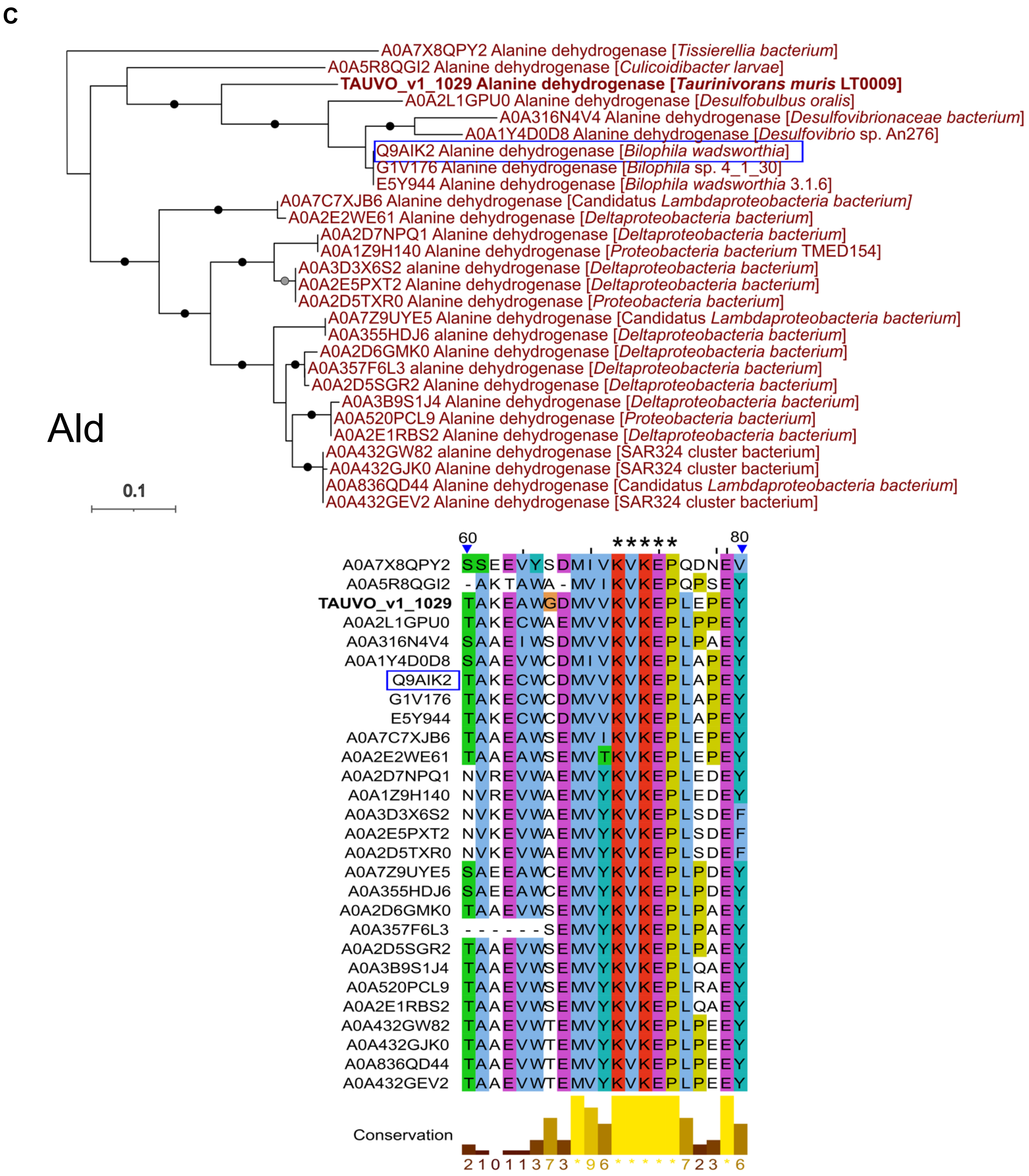

Supplementary Figure S4

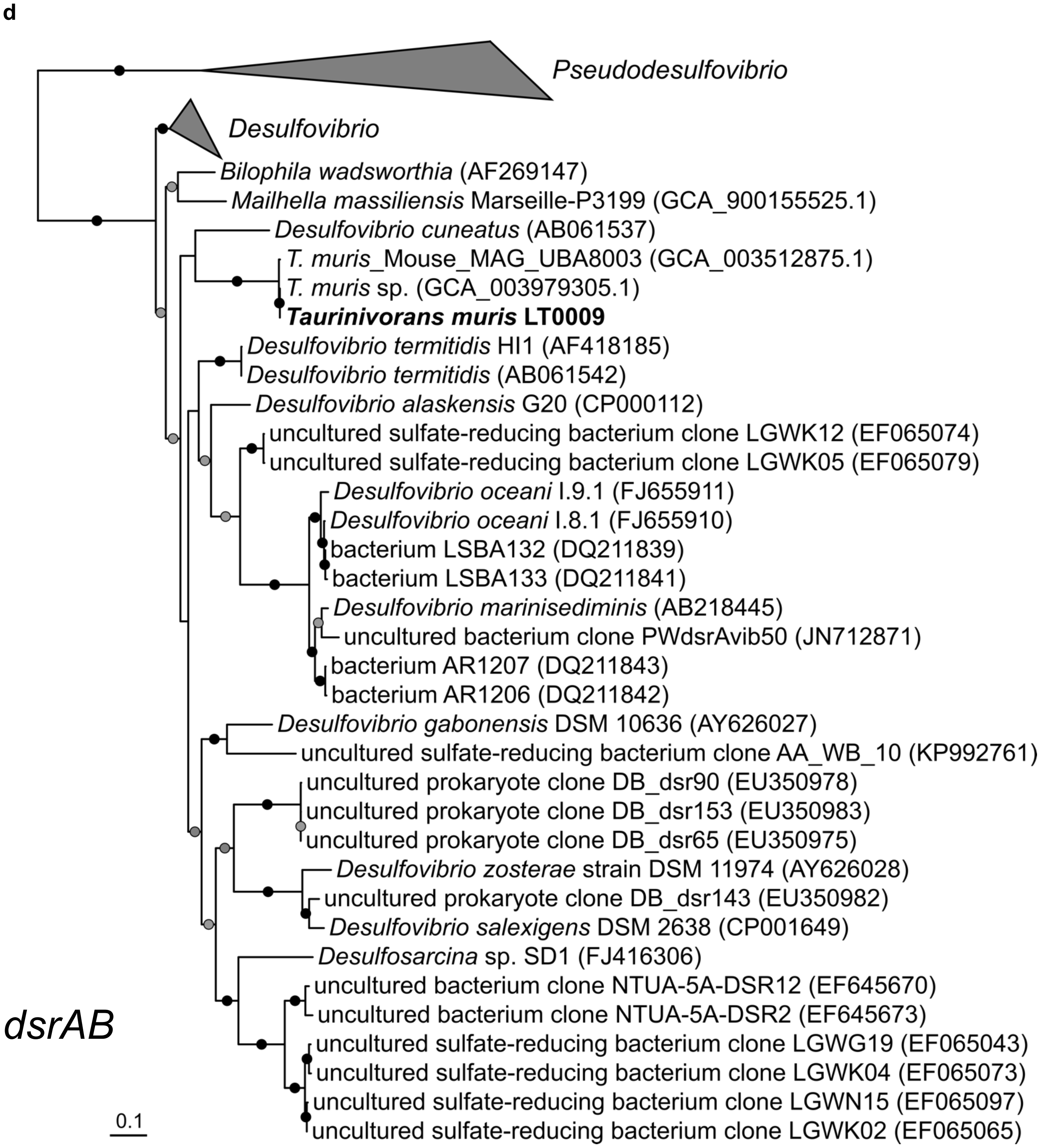

Supplementary Figure S4

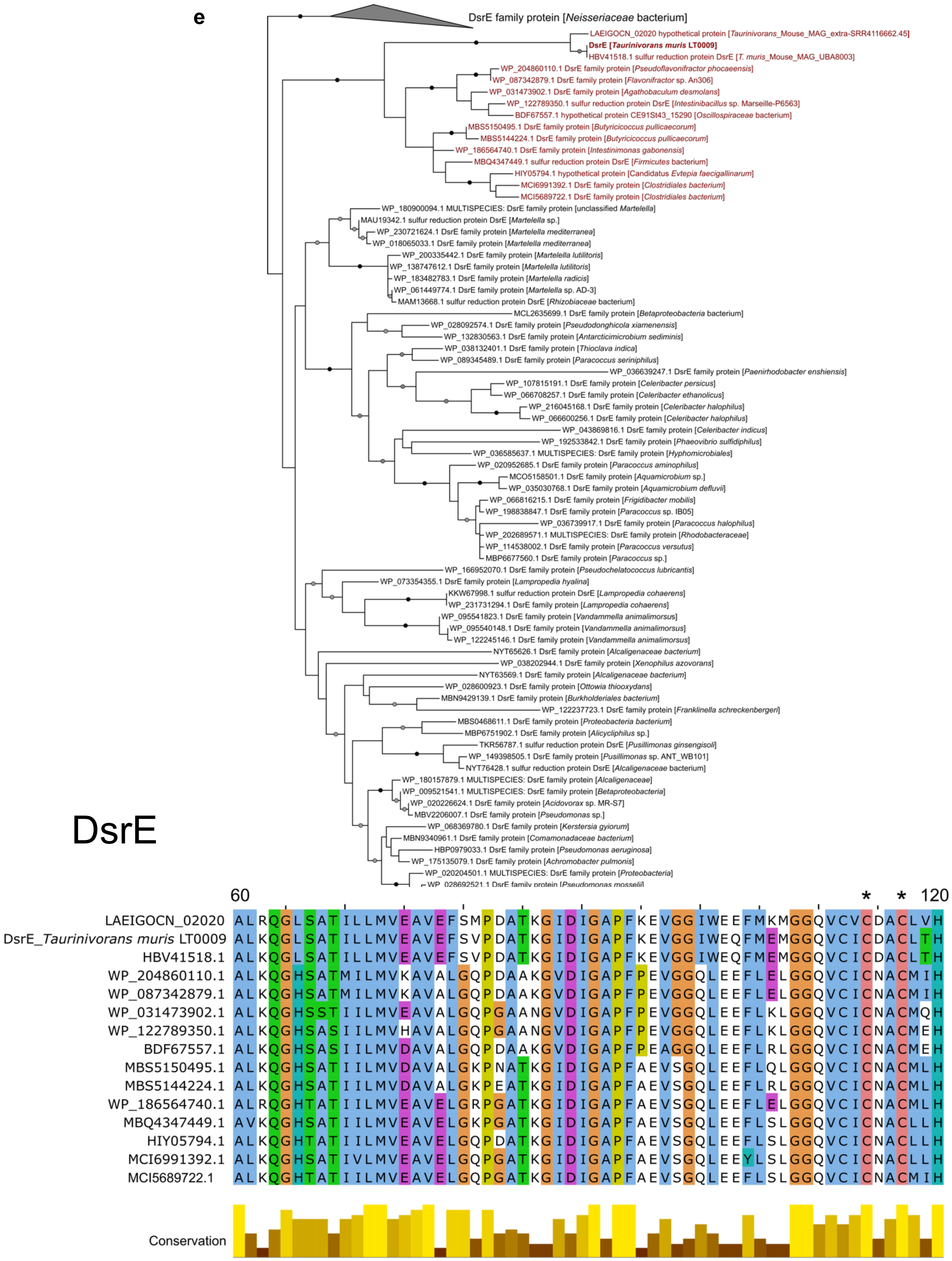

Supplementary Figure S4

f

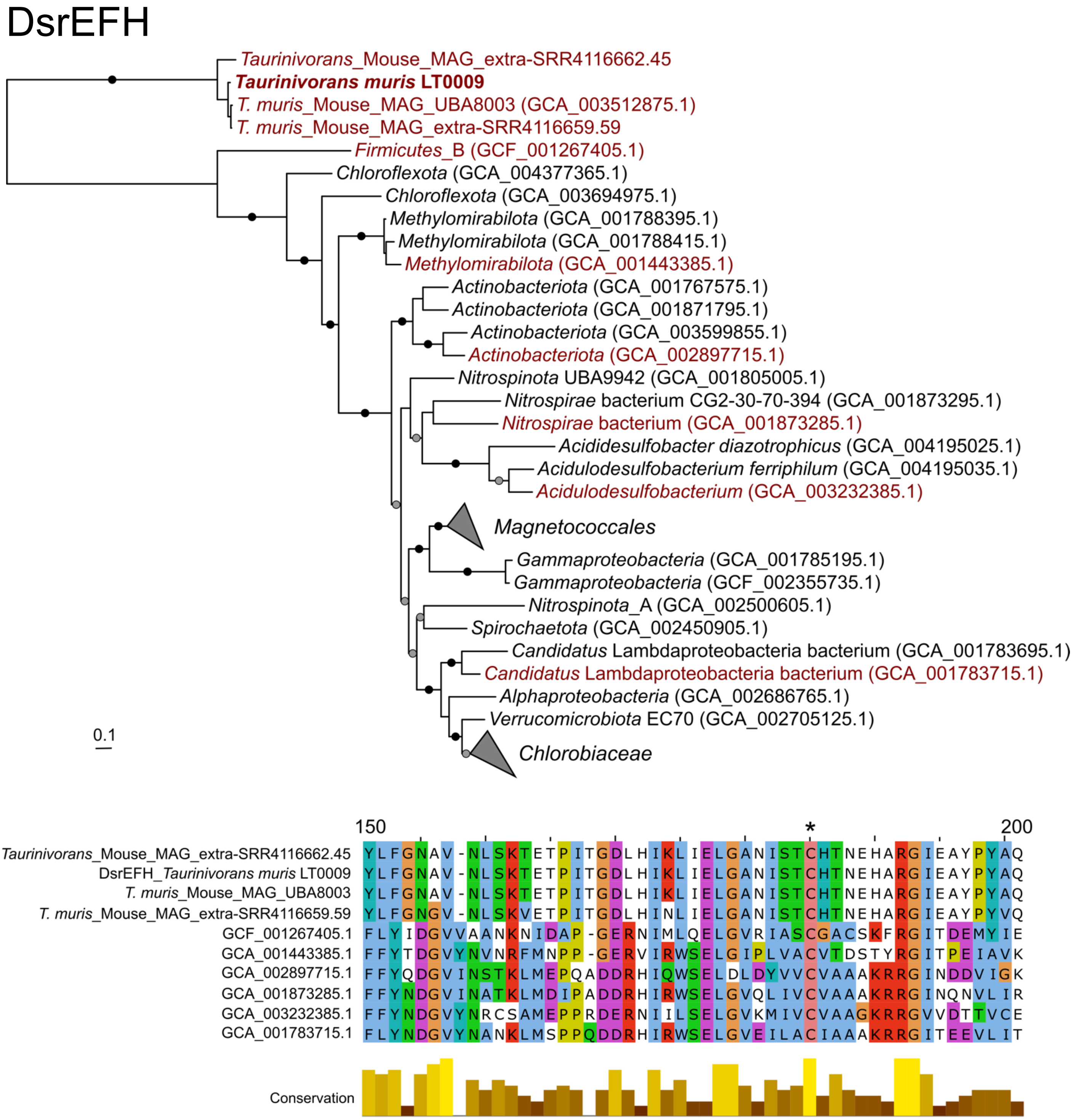

Supplementary Figure S4

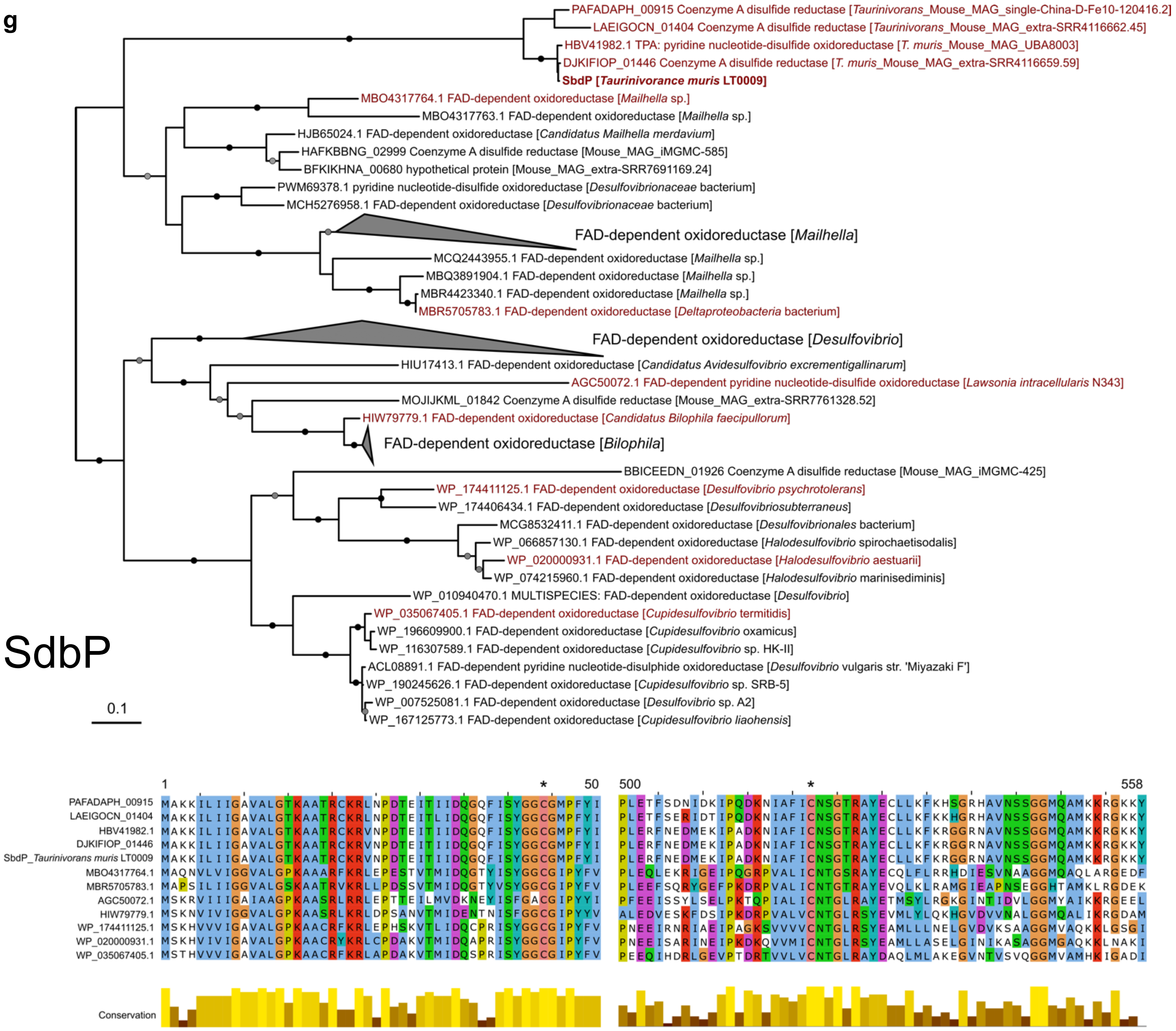

Supplementary Figure 4. Phylogeny and conserved amino acid residues of selected sulfur metabolism proteins of *Taurinivorans muris* LT0009.

a. Tpa protein tree. **b.** Xsc protein tree. **c.** Ald protein tree. **d.** *dsrAB* nucleic acid tree. **e.** DsrE (TAU_v1_1364) protein tree. **f.** Concatenated DsrEFH protein tree. **g.** SdbP protein tree. *Taurinivorans muris* LT0009 is highlighted in bold and the scale bar represents 0.1/1 estimated substitutions per residue in all trees. All trees are midpoint rooted and bootstrap branch supports equal to or greater than 95% and 80% are indicated by black and grey circles, respectively. Amino acid alignments show selected regions with active sites (labeled with an asterisk) conserved across all sequences in the respective tree. The alignment is only shown for sequences marked in red in the tree. The alignment conservation profile is based on all sequences in the tree. The Tpa sequence from *T. muris* is 71% identical to the biochemically characterized Tpa of *B. wadsworthia* RZATAU [82] (marked with a blue box) and 70% identical to Tpa proteins of two other *B. wadsworthia* strains. The Xsc sequence from *T. muris* is most closely related to sequences from other *Desulfovibrionaceae* and shares 61% identity to the biochemically characterized Xsc from *Desulfonispora thiosulfatigenes* (marked with a blue box) [83]. The Ald sequence of *T. muris* shares the highest identity (70%) with the biochemically characterized Ald of *B. wadsworthia* (marked with a blue box) [84]. The inferred DsrE-like amino acid sequence in *T. muris* LT0009 and related sequences contain a Cys-X2-Cys structure different from those in known thiosulfate-transferring DsrE homologs [85]. The cysteine site in DsrEFH is responsible for sulfur atom transfer from DsrEFH to DsrC in sulfur oxidizers [86]. The cysteine in SbdP is also involved in sulfur atom binding [40].

Supplementary Figure S5

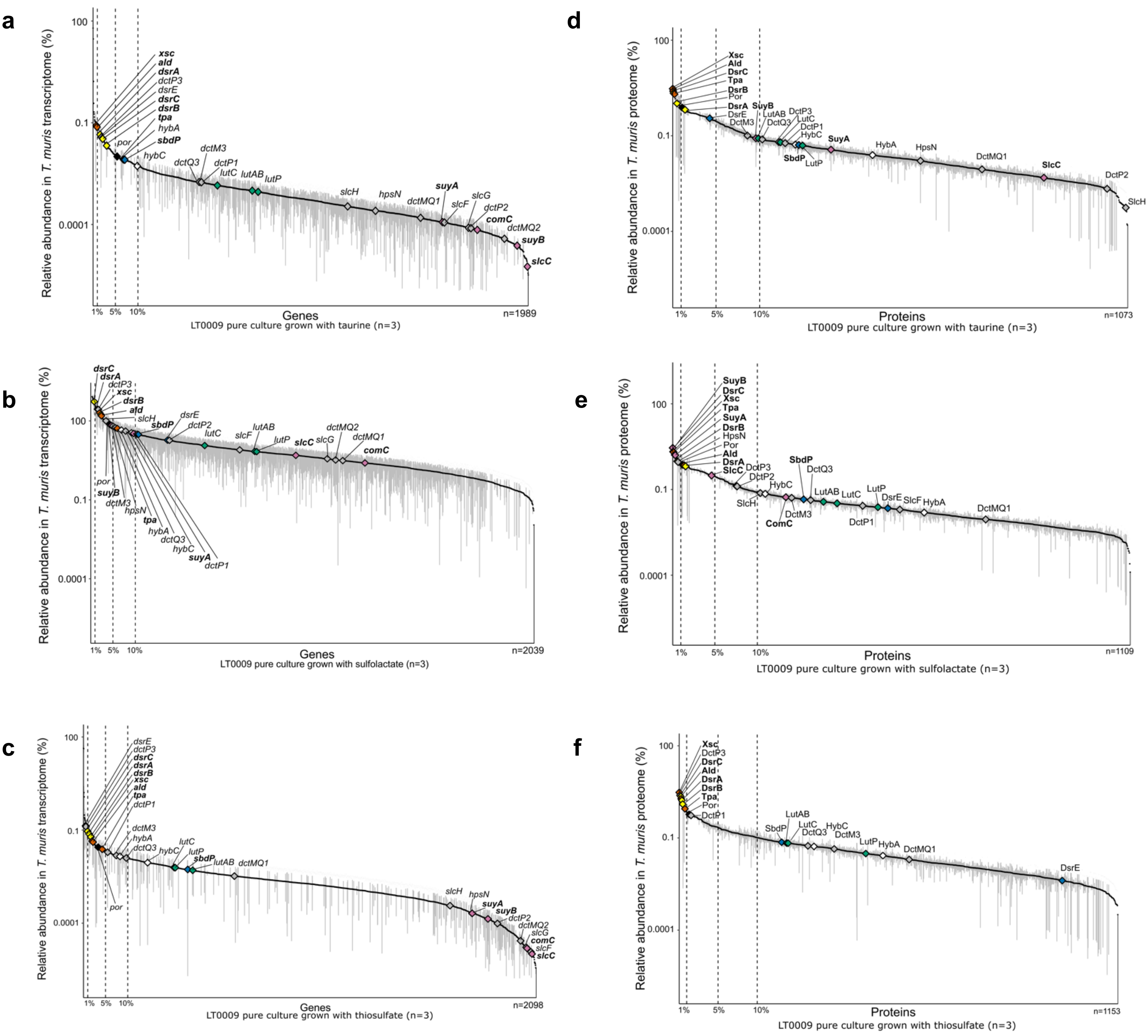

Supplementary Figure 5. **Rank abundance of mRNA expression and protein of the *Taurinivorans muris* LT0009 pure culture grown with taurine, sulfolactate, and thiosulfate.** (**a-c**). Plots show the ranked relative transcript abundance of LT0009 genes and the total number of transcribed LT0009 genes detected in the respective datasets. (**d-f**) Plots show the ranked relative protein abundance of LT0009 proteins and the total number of LT0009 proteins detected in the respective datasets. Genes/proteins for taurine (Tpa, Xsc, Ald), sulfite (DsrAB, DsrC), sulfolactate (SuyAB, SclC, ComC), thiosulfate (SbdP, DsrE), pyruvate (Por), lactate (LutAB, LutC, LuP), and hydrogen (HybA, HybC) metabolism are shown in different colors. Sulfur metabolism genes are further highlighted in bold font. Vertical dashed lines indicate the top 1%, 5%, and 10% of the protein-coding genes in LT0009 genome (n = 2,059). Each point in the rank abundance plot is the mean relative abundance of a gene transcript or a protein and error bars correspond to the 95% confidence interval of the mean. Source data are provided as a Source Data file.

Supplementary Figure S6

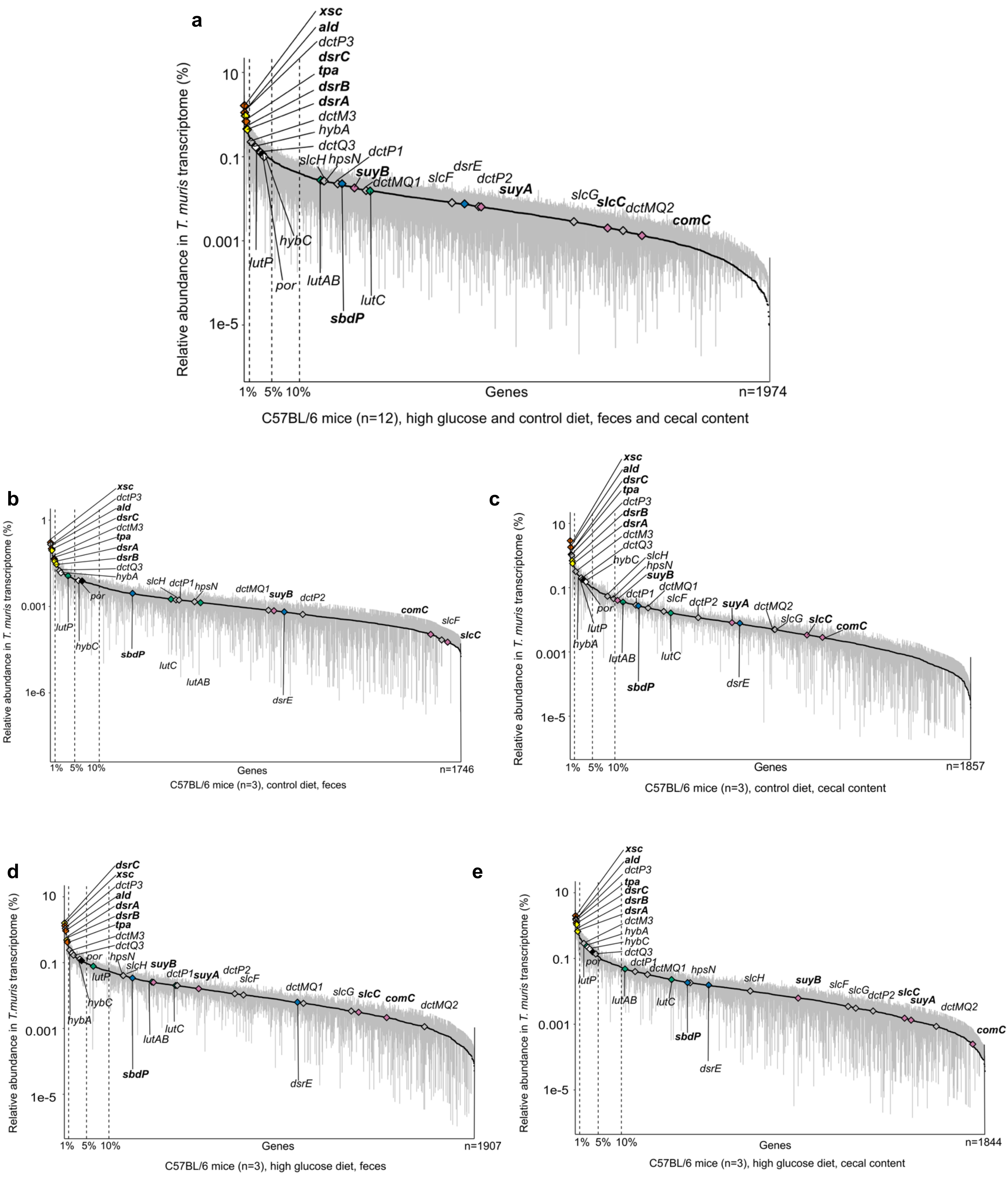

Supplementary Figure S6

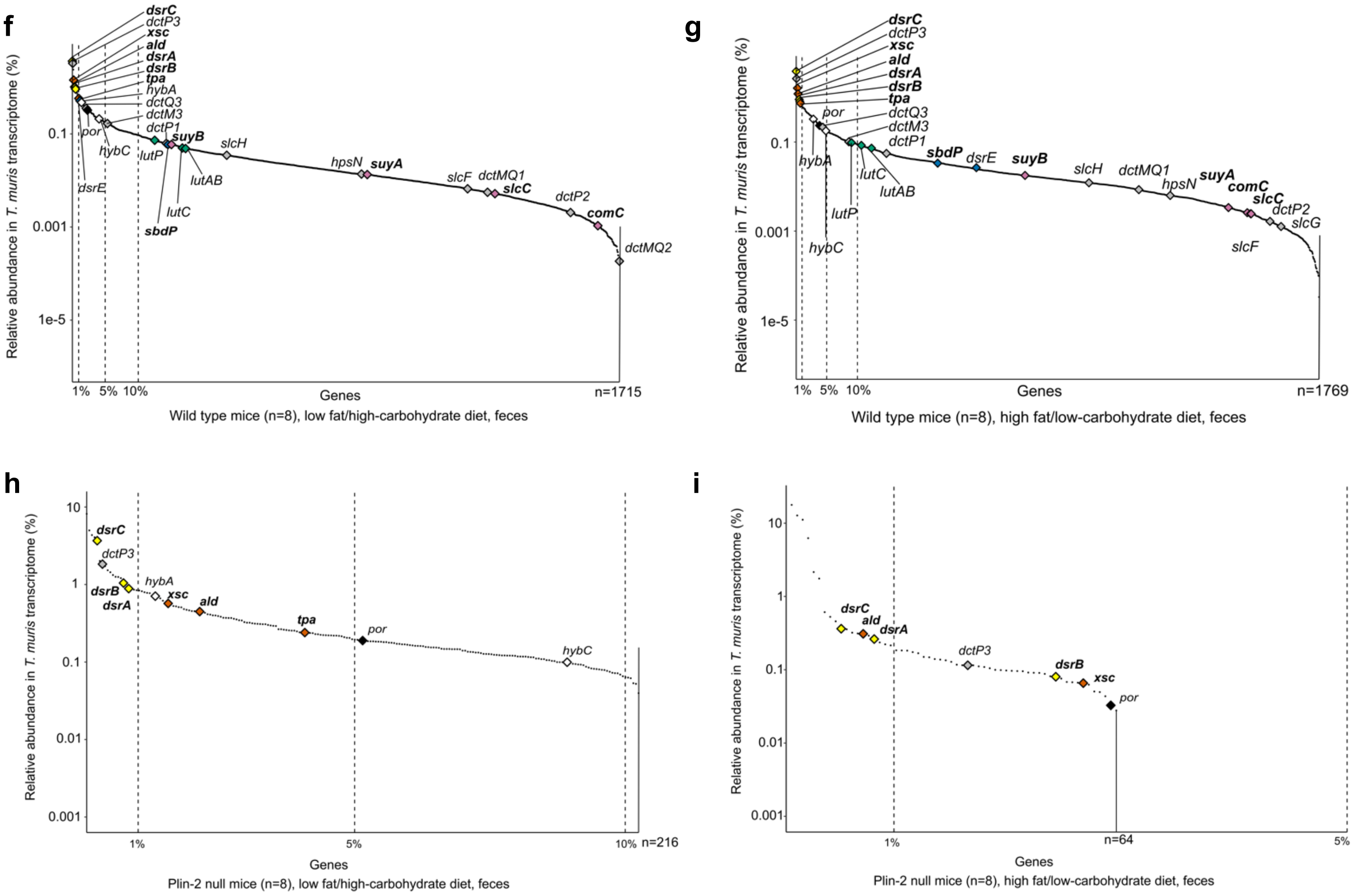

Supplementary Figure 6. Mouse gut metatranscriptomics suggests taurine respiration as the main *in vivo* metabolic niche of *Taurinivorans muris* independent of intestinal location, host genotype, and host diet. LT0009-centric gut metatranscriptome analyses of laboratory mice from two studies: (**a**-**e**) HG study , (**f**-**i**) Plin2 study [61]. All plots show the ranked relative transcript abundance of LT0009 genes and the total number of transcribed LT0009 genes detected in the respective datasets. Genes for taurine (*tpa*, *xsc*, *ald*), sulfite (*dsrAB, dsrC*), sulfolactate (*suyAB*, *sclC*, *comC*), thiosulfate (*sbdP, dsrE*), pyruvate (*por*), lactate (*lutAB, lutC, luP*), and hydrogen (*hybA*, *hybC*) metabolism are shown in different colors. Sulfur metabolism genes are further highlighted in bold font. Vertical dashed lines indicate the top 1%, 5%, and 10% of the protein-coding genes in LT0009 genome (n = 2,059). For the HG study, each point in the rank abundance plot is the mean relative abundance of a gene transcript and error bars correspond to the 95% confidence interval of the mean. The metatranscriptome data from Plin2 study include data from eight animals in one SRA file; data for each individual mouse was not available. Thus, each point in the rank abundance plot represents the relative gene expression across all eight samples. **a.** Gene expressions in all mice from HG study (n = 12). **b.** Gene expressions in fecal samples from mice fed a control diet (n = 3). **c.** Gene expression in cecal samples from mice fed a control diet (n = 3). **d.** Gene expressions in fecal samples from mice fed a high-glucose diet (n = 3). **e.** Gene expressions in cecal samples from mice fed a high-glucose diet (n = 3). **f.** Gene expression in fecal samples from wild type mice fed a low-fat/high-carbohydrate diet (n = 8). **g.** Gene expression in fecal samples from wild type mice fed a high-fat/low-carbohydrate diet (n = 8). **h.** Gene expression in fecal samples from Plin-2 null mice fed a low-fat/high-carbohydrate diet (n = 8). **i.** Gene expression in fecal samples from Plin-2 null mice fed a high-fat/low-carbohydrate diet (n = 8). Source data are provided as a Source Data file.

Supplementary Figure S7

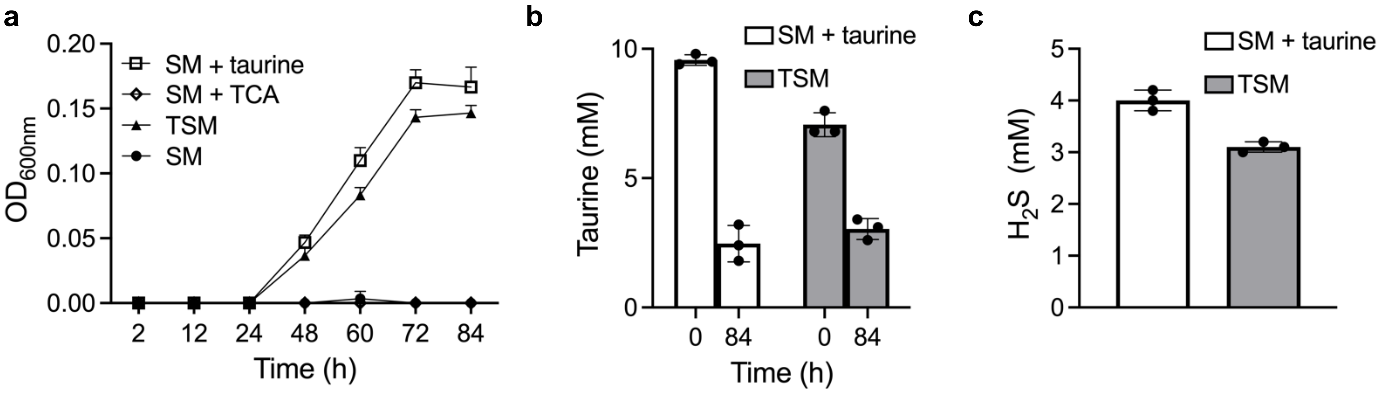

Supplementary Figure 7. Growth experiments with OMM[12] spent medium show that *Taurinivorans muris* LT0009 grows with taurine released from taurocholic acid by other bacteria. a. Growth curves show the averages of optical density measurements at 600 nm (OD_{600nm}) in triplicate cultures. Error bars represent one standard deviation. **b.** Taurine concentrations (mean ± SD) at 0 h and 84 h after inoculation (n = 3 biologically independent cultures). **c.** Hydrogen sulfide concentrations (mean ± SD) at 84 h after inoculation (n = 3 biologically independent cultures). TCA, taurocholic acid; SM, spent medium of OMM[12] strains grown in Anaerobic Akkermansia Medium; TSM, spent medium of OMM[12] strains grown in Anaerobic Akkermansia Medium supplemented with 10 mM TCA; positive control (SM + taurine): SM supplemented with taurine; negative control (SM + TCA): SM supplemented with TCA. Source data are provided as a Source Data file.

Supplementary Figure S8

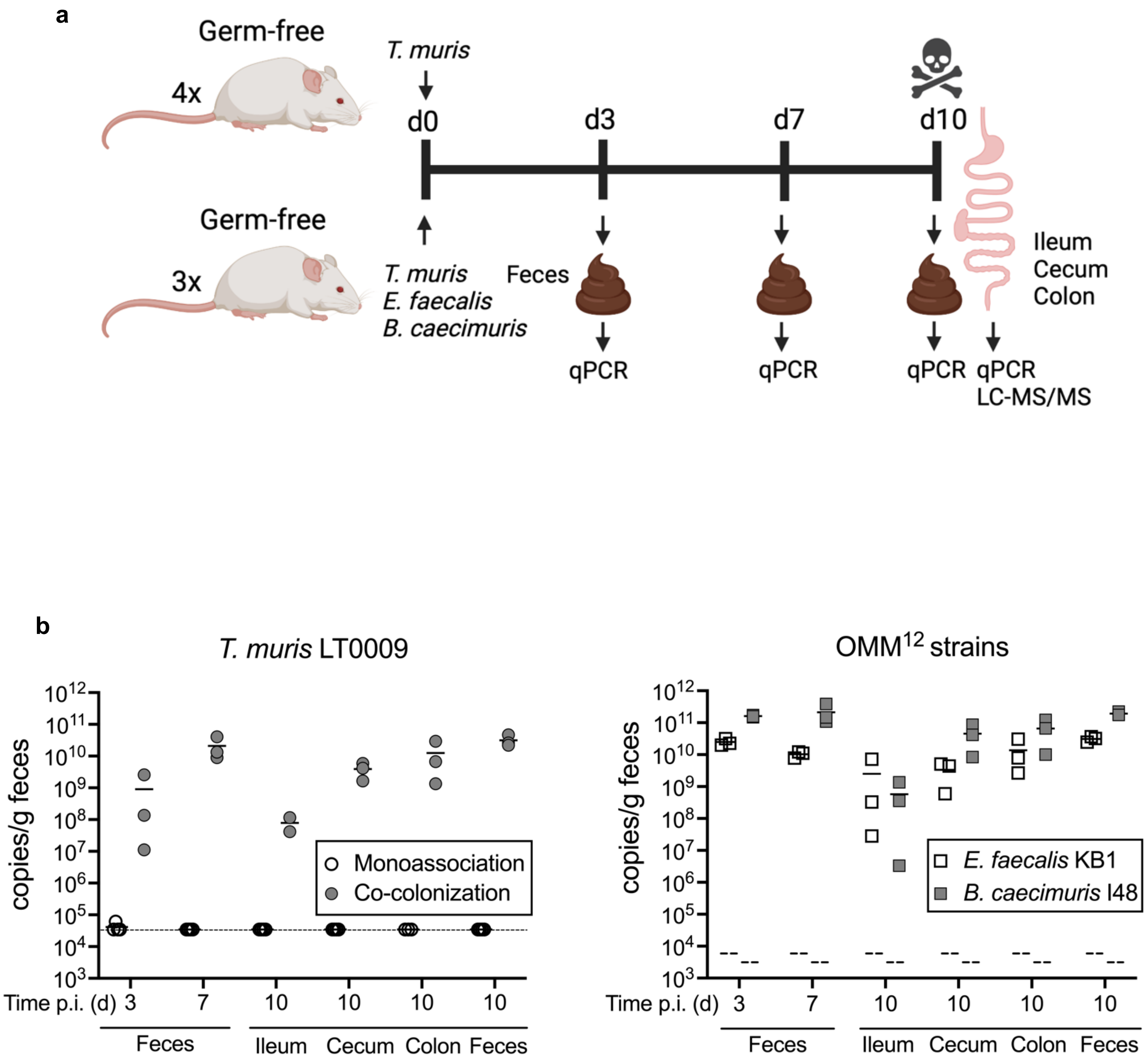

Supplementary Figure S8

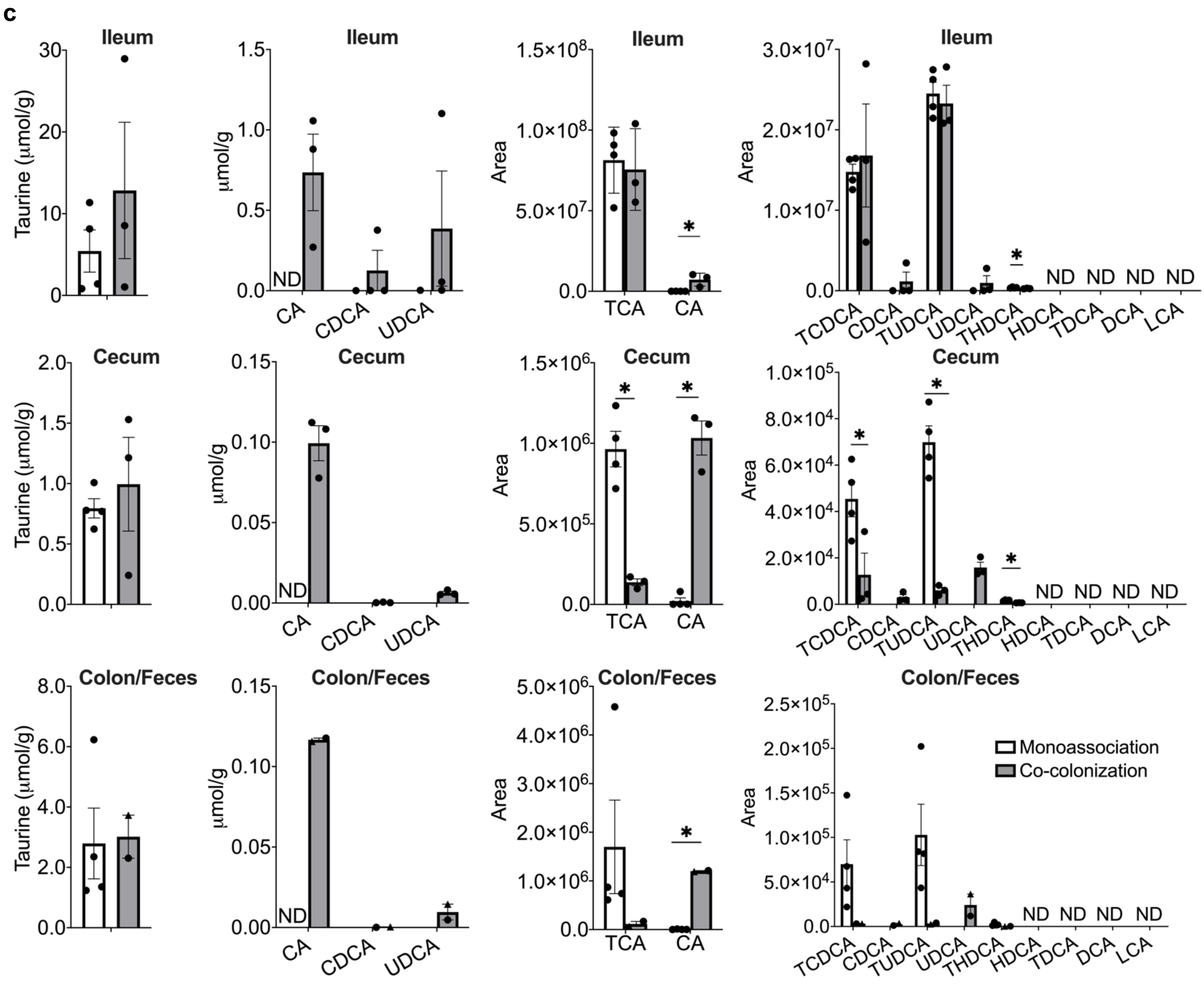

Supplementary Figure 8. The colonization of *Taurinivorans muris* LT0009 in germ-free mice relies on the presence of bacteria that deconjugate taurine-conjugated bile acids. a. Schematic outline of the mono- and co-colonization experiment with germ-free mice. Created with Biorender.com. **b.** Absolute abundances (16S rRNA gene copy numbers per gram feces) of *T. muris* LT0009 (n = 4 monoassociated mice, n = 3 co-colonized mice), *E. faecalis* KB1 (n = 3 co-colonized mice), and *B. caecimuris* I48 (n = 3 co-colonized mice) in ileum, cecum, colon, and feces after inoculation of germ-free mice. Small horizontal lines indicate median values. Dashed horizontal lines indicate the detection limit of strain-specific qPCR assays. **c.** Concentration of taurine and bile acids in ileum, cecum, and colon/feces (n = 4 monoassociated mice, n = 3 co-colonized mice). A colon/feces sample from one co-colonized mice was missing (n = 2). Co-colonized mice exhibited higher CA concentrations in ileum (p = 0.01), cecum (p = 0.0001), and colon/feces ($P < 0.0001$). Taurine-

conjugated bile acids decreased in the ileum (THDCA, $p = 0.04$) and cecum (TCA, $p = 0.002$; TCDCA, $p = 0.04$; TUDCA, $p = 0.001$; THDCA, $p = 0.001$) of co-colonized mice compared to monoassociated mice. Absolute concentrations are expressed in µmol/g and semi-quantitative values are expressed as peak area. Data was analyzed by *t*-test (two-sided) and each data point represents one independent mouse. The fecal sample used for taurine and bile acids measurement is indicated with a triangle. Data presented as mean ± standard error of mean; *$p < 0.05$. CA, cholic acid; CDCA, chenodeoxycholic acid; UDCA, ursodeoxycholic acid; HDCA, hyodeoxycholic acid; DCA, deoxycholic acid; LCA, lithocholic acid; prefix T indicates taurine-conjugated bile acid species; ND, not detected. Source data are provided as a Source Data file.

Supplementary Figure S9

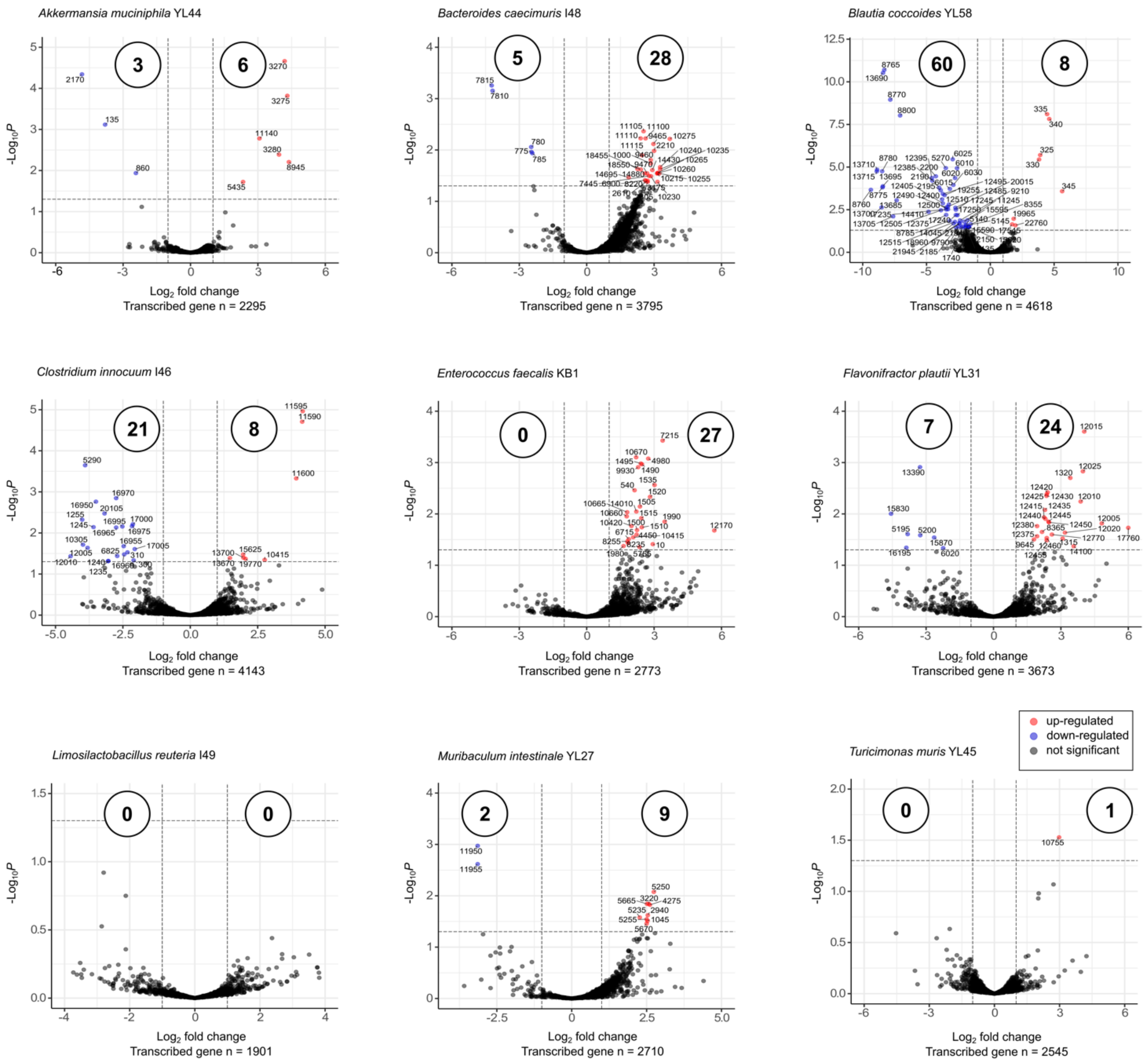

Supplementary Figure 9. Differential gene expression of OMM^{12} strains in gnotobiotic mice with and without *Taurinivorans muris* LT0009 and infected with *Salmonella enterica* Tm^{avir} M2702. No reads with 98% sequence identity to genomes of *A. muris* KB18 and *B. animalis* YL2 were detected, which is consistent with lack of evidence of colonization of these strains by qPCR (Fig. 4b). Volcano plots show differential gene transcription of individual OMM^{12} strains in mice with (n = 3 mice) and without LT0009 (n = 3 mice). Differential genes were detected using DeSeq2 (Wald test, 5% false discovery rate). The x-axis shows log-fold-change in transcription and the y-axis shows the negative logarithm10-transformed adjusted p values. Red and blue dots show significantly (adjusted-p value <0.05) up-regulated (log2 fold change >1) and down-regulated (log2 fold change <-1) genes in mice with LT0009, respectively, and are labeled with locus tag numbers. Numbers in circles show the total numbers of up- and down-regulated genes per strain.

Supplementary Figure S10

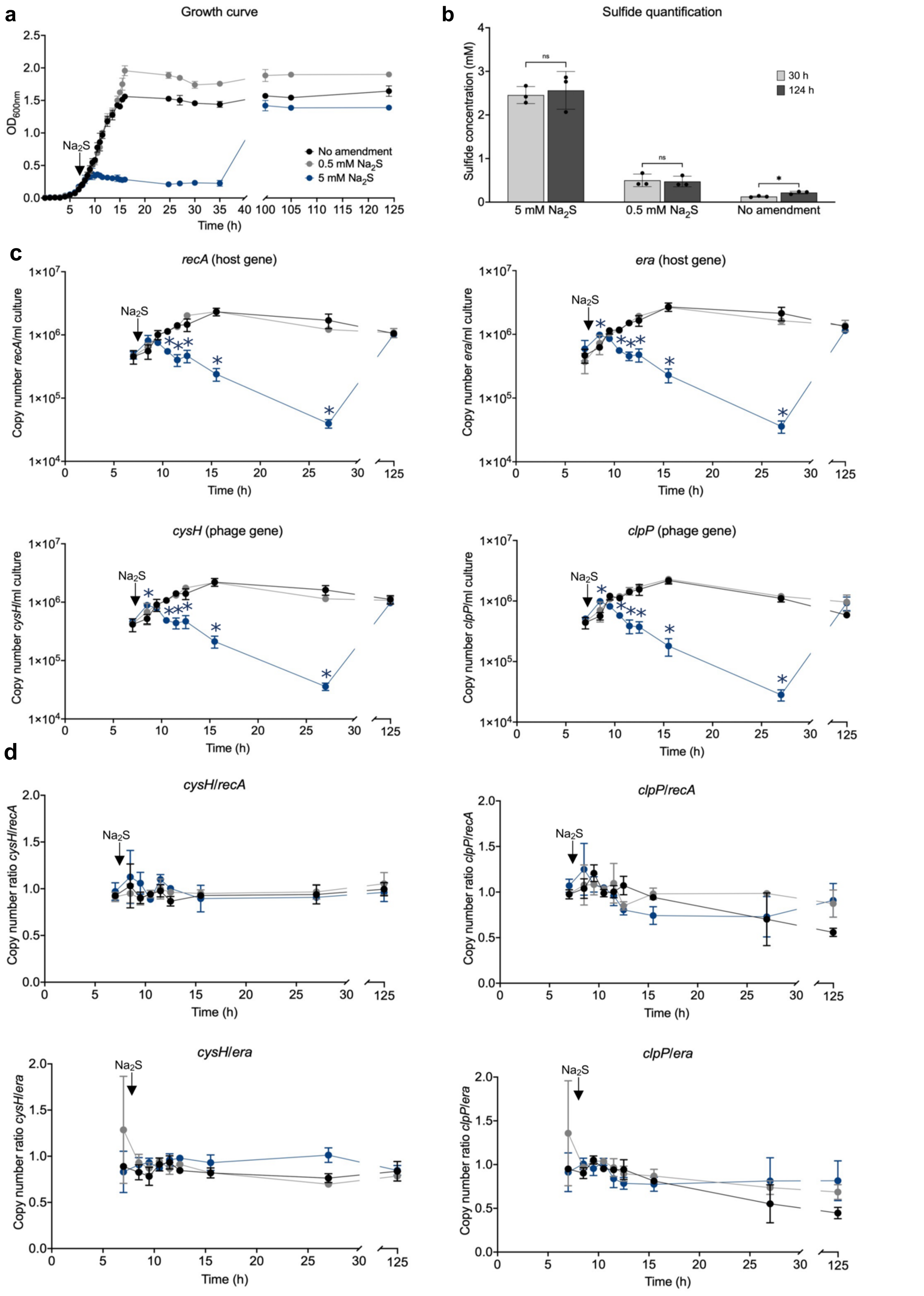

Supplementary Figure 10. Impact of sulfide on growth of *Enterocloster clostridioformis* YL32 and activation of its prophage Saumur a. Optical density (OD) of *E. clostridioformis* YL32 cultures grown with (0.5 mM, 5 mM) Na_2S. Arrow indicates when Na_2S was added to the cultures (n = 3 biologically independent cultures). Data shown as mean OD_{600nm} ± standard deviation. The growth curve of the unamended control is depicted in black. The growth curves of the 0.5 mM Na_2S treatment and the 5 mM Na_2S treatment are depicted in gray and blue, respectively. Coloring also applies to panels c and d. **b.** Sulfide concentrations at 30 h and 124 h (n = 3 biologically independent cultures). Data are presented as mean ± standard deviation. Results were statistically analyzed using paired t-tests, p value adjusted with Holm-Sidak method. Asterisk indicates significant difference of sulfide concentration in no-amendment cultures between 30 h and 120 h (adjusted p = 0.04). **c.** Copy numbers per ml culture of two host genes (*recA*, *era*) and two genes of the prophage Saumur (*cysH*, *clpP*) were quantified using ddPCR (n=3 biologically independent cultures). Plots show the mean copy numbers of the respective genes ± standard deviation. Arrows indicate when Na_2S was added to the cultures. Statistical analysis was performed using two-way ANOVA and subsequent Tukey's Honest Significant Difference post-hoc test. Significant differences between treatments and the unamended control (adjusted p-value < 0.05) for the individual time points are marked with an asterisk. **d.** Copy number ratio of phage gene to host gene for all four combinations. Data are presented as mean ± standard deviation. A phage-to-host gene ratio significantly higher than one would indicate activation of the prophage [102,103]. Arrows indicate when Na_2S was added to the cultures. Source data are provided as a Source Data file.

Supplementary References

1. Laue, H. & Cook, A. M. Biochemical and molecular characterization of taurine:pyruvate aminotransferase from the anaerobe Bilophila wadsworthia. *Eur. J. Biochem.* **267**, 6841–6848 (2000).
2. Laue, H., Denger, K. & Cook, A. M. Taurine reduction in anaerobic respiration of Bilophila wadsworthia RZATAU. *Appl. Environ. Microbiol.* **63**, 2016–2021 (1997).
3. da Silva, S. M., Venceslau, S. S., Fernandes, C. L. V., Valente, F. M. A. & Pereira, I. A. C. Hydrogen as an energy source for the human pathogen Bilophila wadsworthia. *Antonie Van Leeuwenhoek* **93**, 381–390 (2008).
4. Roy, A. B., Hewlins, M. J. E., Ellis, A. J., Harwood, J. L. & White, G. F. Glycolytic breakdown of sulfoquinovose in bacteria: a missing link in the sulfur cycle. *Appl. Environ. Microbiol.* **69**, 6434–6441 (2003).
5. Mayer, J. *et al.* 2,3-Dihydroxypropane-1-sulfonate degraded by Cupriavidus pinatubonensis JMP134: purification of dihydroxypropanesulfonate 3-dehydrogenase. *Microbiology* **156**, 1556–1564 (2010).
6. Wang, X., Chi, D., Su, G., Li, L. & Shao, L. Determination of taurine in biological samples by high-performance liquid chromatography using 4-fluoro-7-nitrobenzofurazan as a derivatizing agent. *Biomed. Environ. Sci.* **24**, 537–542 (2011).
7. Cline, J. D. Spectrophotometric determination of hydrogen sulfide in natural water. *Limnology and Oceanography* **14**, 454–458 (1969).
8. Daims, H., Stoecker, K. & Wagner, M. Fluorescence in situ hybridization for the detection of prokaryotes. in *Molecular Microbial Ecology* (eds. Osborn M. & Smith C. 208–228 (Taylor & Francis, 2004).
9. Riva, A. *et al.* A fiber-deprived diet disturbs the fine-scale spatial architecture of the murine colon microbiome. *Nat. Commun.* **10**, 4366 (2019).
10. Daims, H., Lücker, S. & Wagner, M. daime, a novel image analysis program for microbial ecology and biofilm research. *Environ. Microbiol.* **8**, 200–213 (2006).
11. Yilmaz, P. *et al.* The SILVA and 'All-species Living Tree Project (LTP)' taxonomic frameworks. *Nucleic Acids Research* **42**, D643–D648 (2014).
12. Ludwig, W. *et al.* ARB: a software environment for sequence data. *Nucleic Acids Res.* **32**, 1363–1371 (2004).
13. Quast, C. *et al.* The SILVA ribosomal RNA gene database project: improved data processing and web-based tools. *Nucleic Acids Res.* **41**, D590–6 (2013).
14. Wick, R. R., Judd, L. M., Gorrie, C. L. & Holt, K. E. Unicycler: Resolving bacterial genome assemblies from short and long sequencing reads. *PLOS Computational Biology* **13**, e1005595 (2017).
15. Vallenet, D. *et al.* MicroScope in 2017: an expanding and evolving integrated resource for community expertise of microbial genomes. *Nucleic Acids Res.* **45**, D517–D528 (2017).
16. Hausmann, B., Pelikan, C., Rattei, T., Loy, A. & Pester, M. Long-Term Transcriptional Activity at Zero Growth of a Cosmopolitan Rare Biosphere Member. *MBio* **10**, (2019).
17. Famiglietti, M. L. *et al.* Genetic variations and diseases in UniProtKB/Swiss-Prot: the ins and outs of expert manual curation. *Hum. Mutat.* **35**, 927–935 (2014).
18. Kersey, P., Hermjakob, H. & Apweiler, R. VARSPLIC: alternatively-spliced protein sequences derived from SWISS-PROT and TrEMBL. *Bioinformatics* **16**, 1048–1049 (2000).
19. Søndergaard, D., Pedersen, C. N. S. & Greening, C. HydDB: A web tool for hydrogenase classification

and analysis. *Sci. Rep.* **6**, 34212 (2016).
20. Johnson, M. *et al.* NCBI BLAST: a better web interface. *Nucleic Acids Res.* **36**, W5–W9 (2008).
21. Parte, A. C. LPSN - List of Prokaryotic names with Standing in Nomenclature (bacterio.net), 20 years on. *Int. J. Syst. Evol. Microbiol.* **68**, 1825–1829 (2018).
22. Edgar, R. C. MUSCLE: multiple sequence alignment with high accuracy and high throughput. *Nucleic Acids Res.* **32**, 1792–1797 (2004).
23. Capella-Gutiérrez, S., Silla-Martínez, J. M. & Gabaldón, T. trimAl: a tool for automated alignment trimming in large-scale phylogenetic analyses. *Bioinformatics* **25**, 1972–1973 (2009).
24. Nguyen, L.-T., Schmidt, H. A., von Haeseler, A. & Minh, B. Q. IQ-TREE: a fast and effective stochastic algorithm for estimating maximum-likelihood phylogenies. *Mol. Biol. Evol.* **32**, 268–274 (2015).
25. Hoang, D. T., Chernomor, O., von Haeseler, A., Minh, B. Q. & Vinh, L. S. UFBoot2: Improving the Ultrafast Bootstrap Approximation. *Mol. Biol. Evol.* **35**, 518–522 (2018).
26. Eddy, S. R. Accelerated Profile HMM Searches. *PLoS Comput. Biol.* **7**, e1002195 (2011).
27. Mistry, J. *et al.* Pfam: The protein families database in 2021. *Nucleic Acids Res.* **49**, D412–D419 (2021).
28. Paysan-Lafosse, T. *et al.* InterPro in 2022. *Nucleic Acids Res.* **51**, D418–D427 (2023).
29. Haft, D. H., Selengut, J. D. & White, O. The TIGRFAMs database of protein families. *Nucleic Acids Res.* **31**, 371–373 (2003).
30. Huerta-Cepas, J. *et al.* eggNOG 5.0: a hierarchical, functionally and phylogenetically annotated orthology resource based on 5090 organisms and 2502 viruses. *Nucleic Acids Res.* **47**, D309–D314 (2019).
31. Katoh, K. MAFFT: a novel method for rapid multiple sequence alignment based on fast Fourier transform. *Nucleic Acids Research* **30**, 3059–3066 (2002).
32. Trifinopoulos, J., Nguyen, L.-T., von Haeseler, A. & Minh, B. Q. W-IQ-TREE: a fast online phylogenetic tool for maximum likelihood analysis. *Nucleic Acids Res.* **44**, W232–5 (2016).
33. Letunic, I. & Bork, P. Interactive tree of life (iTOL) v3: an online tool for the display and annotation of phylogenetic and other trees. *Nucleic Acids Res.* **44**, W242–5 (2016).
34. Lesker, T. R. *et al.* An Integrated Metagenome Catalog Reveals New Insights into the Murine Gut Microbiome. *Cell Rep.* **30**, 2909–2922.e6 (2020).
35. Parks, D. H., Imelfort, M., Skennerton, C. T., Hugenholtz, P. & Tyson, G. W. CheckM: assessing the quality of microbial genomes recovered from isolates, single cells, and metagenomes. *Genome Res.* **25**, 1043–1055 (2015).
36. Rodriguez-R, L. M. & Konstantinidis, K. T. The enveomics collection: a toolbox for specialized analyses of microbial genomes and metagenomes. Preprint at https://peerj.com/preprints/1900 (2016).
37. Jain, C., Rodriguez-R, L. M., Phillippy, A. M., Konstantinidis, K. T. & Aluru, S. High throughput ANI analysis of 90K prokaryotic genomes reveals clear species boundaries. *Nat. Commun.* **9**, 5114 (2018).
38. Hanson, B. T. *et al.* Sulfoquinovose is a select nutrient of prominent bacteria and a source of hydrogen sulfide in the human gut. *ISME J.* **15**, 1–13 (2021).
39. Heinzinger, N. K., Fujimoto, S. Y., Clark, M. A., Moreno, M. S. & Barrett, E. L. Sequence analysis of the phs operon in Salmonella typhimurium and the contribution of thiosulfate reduction to anaerobic energy metabolism. *J. Bacteriol.* **177**, 2813–2820 (1995).
40. Aketagawa, J., Kobayashi, K. & Ishimoto, M. Purification and properties of thiosulfate reductase from Desulfovibrio vulgaris, Miyazaki F. *J. Biochem.* **97**, 1025–1032 (1985).
41. Rameez, M. J. *et al.* Two pathways for thiosulfate oxidation in the alphaproteobacterial chemolithotroph Paracoccus thiocyanatus SST. *Microbiol. Res.* **230**, 126345 (2020).

42. Koch, T. & Dahl, C. A novel bacterial sulfur oxidation pathway provides a new link between the cycles of organic and inorganic sulfur compounds. *ISME J.* **12**, 2479–2491 (2018).
43. Aussignargues, C. *et al.* Rhodanese functions as sulfur supplier for key enzymes in sulfur energy metabolism. *J. Biol. Chem.* **287**, 19936–19948 (2012).
44. Zhang, J. *et al.* A novel bacterial thiosulfate oxidation pathway provides a new clue about the formation of zero-valent sulfur in deep sea. *ISME J.* **14**, 2261–2274 (2020).
45. Peck, S. C. *et al.* A glycyl radical enzyme enables hydrogen sulfide production by the human intestinal bacterium Bilophila wadsworthia. *Proceedings of the National Academy of Sciences* **116,** 3171–3176 (2019).
46. BBMap. SourceForge https://sourceforge.net/projects/bbmap/.
47. Li, B., Ruotti, V., Stewart, R. M., Thomson, J. A. & Dewey, C. N. RNA-Seq gene expression estimation with read mapping uncertainty. *Bioinformatics* **26**, 493–500 (2010).
48. Love, M. I., Huber, W. & Anders, S. Moderated estimation of fold change and dispersion for RNA-seq data with DESeq2. *Genome Biol.* **15**, 550 (2014).
49. Lagkouvardos, I. *et al.* IMNGS: A comprehensive open resource of processed 16S rRNA microbial profiles for ecology and diversity studies. *Sci. Rep.* **6**, 33721 (2016).
50. Angel, R., Claus, P. & Conrad, R. Methanogenic archaea are globally ubiquitous in aerated soils and become active under wet anoxic conditions. *ISME J.* **6**, 847–862 (2012).
51. Xiong, X. *et al.* Perilipin-2 modulates dietary fat-induced microbial global gene expression profiles in the mouse intestine. *Microbiome* **5**, 117 (2017).
52. Stacy, A. *et al.* Infection trains the host for microbiota-enhanced resistance to pathogens. *Cell* **184**, 615–627.e17 (2021).
53. Rosshart, S. P. *et al.* Wild Mouse Gut Microbiota Promotes Host Fitness and Improves Disease Resistance. *Cell* **171**, 1015–1028.e13 (2017).
54. Parks, D. H. *et al.* GTDB: an ongoing census of bacterial and archaeal diversity through a phylogenetically consistent, rank normalized and complete genome-based taxonomy. *Nucleic Acids Res.* **50**, D785–D794 (2022).
55. Bushnell B. BBMap short read aligner, and other bioinformatic tools. sourceforge.net/projects/bbmap/ (2019).
56. Callahan, B. J. *et al.* DADA2: High-resolution sample inference from Illumina amplicon data. *Nat. Methods* **13**, 581–583 (2016).
57. Eberl, C. *et al.* Reproducible Colonization of Germ-Free Mice With the Oligo-Mouse-Microbiota in Different Animal Facilities. *Front. Microbiol.* **10**, 2999 (2019).
58. Brugiroux, S. *et al.* Genome-guided design of a defined mouse microbiota that confers colonization resistance against Salmonella enterica serovar Typhimurium. *Nat Microbiol* **2**, 16215 (2016).
59. Herp, S. *et al.* Mucispirillum schaedleri Antagonizes Salmonella Virulence to Protect Mice against Colitis. *Cell Host Microbe* **25**, 681–694.e8 (2019).
60. Bustin, S. A. *et al.* The MIQE Guidelines: Minimum Information for Publication of Quantitative Real-Time PCR Experiments. *Clinical Chemistry* **55,** 611–622 (2009).
61. Lamy-Besnier, Q., Koszul, R., Debarbieux, L. & Marbouty, M. Closed and High-Quality Bacterial Genome Sequences of the Oligo-Mouse-Microbiota Community. *Microbiol Resour Announc* **10**, 17 e01396-20 (2021).
62. Song, Z. *et al.* Taxonomic profiling and populational patterns of bacterial bile salt hydrolase (BSH) genes based on worldwide human gut microbiome. *Microbiome* **7**, 9 (2019).

63. Arndt, D. *et al.* PHASTER: a better, faster version of the PHAST phage search tool. *Nucleic Acids Res.* **44**, W16–21 (2016).
64. Pristner, M. *et al.* Neuroactive metabolites and bile acids are altered in extremely premature infants with brain injury. Preprint at https://doi.org/10.1101/2023.05.17.23290088.
65. Adams, K. J. *et al.* Skyline for Small Molecules: A Unifying Software Package for Quantitative Metabolomics. *J. Proteome Res.* **19**, 1447–1458 (2020).
66. Dutra, L., Franz, O., Puupponen, V.-M. & Tiirola, M. DNA recovery from Droplet Digital^{TM} PCR emulsions using liquid nitrogen. *Biotechniques* **69**, 450–454 (2020).
67. Chai, Y., Kolter, R. & Losick, R. A Widely Conserved Gene Cluster Required for Lactate Utilization in Bacillus subtilis and Its Involvement in Biofilm Formation. *Journal of Bacteriology* **191,** 2423–2430 (2009).
68. Pinchuk, G. E. *et al.* Genomic reconstruction of Shewanella oneidensis MR-1 metabolism reveals a previously uncharacterized machinery for lactate utilization. *Proc. Natl. Acad. Sci. U. S. A.* **106**, 2874–2879 (2009).
69. Thomas, M. T. *et al.* Two respiratory enzyme systems in Campylobacter jejuni NCTC 11168 contribute to growth on L-lactate. *Environ. Microbiol.* **13**, 48–61 (2011).
70. Laue, H., Denger, K. & Cook, A. M. Taurine reduction in anaerobic respiration of Bilophila wadsworthia RZATAU. *Applied and environmental microbiology* **63**, 2016–2021 (1997).
71. Wolf, P. G., Biswas, A., Morales, S. E., Greening, C. & Gaskins, H. R. H2 metabolism is widespread and diverse among human colonic microbes. *Gut Microbes* **7**, 235–245 (2016).
72. Hopper, C. P. *et al.* Role of Carbon Monoxide in Host–Gut Microbiome Communication. *Chemical Reviews* **120**, 13273–13311 (2020).
73. Diender, M., Stams, A. J. M. & Sousa, D. Z. Pathways and Bioenergetics of Anaerobic Carbon Monoxide Fermentation. *Front. Microbiol.* **6**, 1275 (2015).
74. Singer S.W., Hirst M.B. & Ludden P.W. CO-dependent H2 evolution by Rhodospirillum rubrum: Role of CODH:CooF complex. *Biochimica et Biophysica Acta (BBA) - Bioenergetics* **1757**, 1582–1591 (2006).
75. Kuhns, M., Trifunović, D., Huber, H. & Müller, V. The Rnf complex is a Na coupled respiratory enzyme in a fermenting bacterium, Thermotoga maritima. *Communications Biology* **3**, (2020).
76. Lamy-Besnier, Q. *et al.* Chromosome folding and prophage activation reveal specific genomic architecture for intestinal bacteria. *Microbiome* **11**, 111 (2023).
77. Zünd, M. *et al.* High throughput sequencing provides exact genomic locations of inducible prophages and accurate phage-to-host ratios in gut microbial strains. *Microbiome* **9**, 77 (2021).
78. Kieft, K. *et al.* Virus-associated organosulfur metabolism in human and environmental systems. *Cell Rep.* **36**, 109471 (2021).
79. Hu, J., Ye, H., Wang, S., Wang, J. & Han, D. Prophage Activation in the Intestine: Insights Into Functions and Possible Applications. *Front. Microbiol.* **12**, 785634 (2021).
80. Otsuji, N., Sekiguchi, M., Iijima, T. & Takagi, Y. Induction of phage formation in the lysogenic Escherichia coli K-12 by mitomycin C. *Nature* **184 (Suppl 14)**, 1079–1080 (1959).
81. Tomasova, L., Konopelski, P. & Ufnal, M. Gut Bacteria and Hydrogen Sulfide: The New Old Players in Circulatory System Homeostasis. *Molecules* **21**, (2016).
82. Dordević, D., Jančíková, S., Vítězová, M. & Kushkevych, I. Hydrogen sulfide toxicity in the gut environment: Meta-analysis of sulfate-reducing and lactic acid bacteria in inflammatory processes. *J. Advert. Res.* **27**, 55–69 (2021).
83. Magee, E. A., Richardson, C. J., Hughes, R. & Cummings, J. H. Contribution of dietary protein to sulfide

production in the large intestine: an in vitro and a controlled feeding study in humans. *Am. J. Clin. Nutr.* **72**, 1488–1494 (2000).

84. Deplancke, B. *et al.* Gastrointestinal and microbial responses to sulfate-supplemented drinking water in mice. *Exp. Biol. Med.* **228**, 424–433 (2003).
85. Levitt, M. D., Springfield, J., Furne, J., Koenig, T. & Suarez, F. L. Physiology of sulfide in the rat colon: use of bismuth to assess colonic sulfide production. *J. Appl. Physiol.* **92**, 1655–1660 (2002).
86. Pérez Escriva, P., Fuhrer, T. & Sauer, U. Distinct N and C Cross-Feeding Networks in a Synthetic Mouse Gut Consortium. *mSystems* **7**, e0148421 (2022).
87. Ridlon, J. M., Wolf, P. G. & Gaskins, H. R. Taurocholic acid metabolism by gut microbes and colon cancer. *Gut Microbes* **7**, 201–215 (2016).
88. Streidl, T. *et al.* The gut bacterium produces secondary bile acids and influences liver physiology in gnotobiotic mice. *Gut Microbes* **13**, 1–21 (2021).
89. Huxtable, R. J. & Lippincott, S. E. Diet and biosynthesis as sources of taurine in the mouse. *J. Nutr.* **112**, 1003–1010 (1982).
90. Swann, J. R. *et al.* Systemic gut microbial modulation of bile acid metabolism in host tissue compartments. *Proc. Natl. Acad. Sci. U. S. A.* **108 Suppl 1**, 4523–4530 (2011).
91. Sayin, S. I. *et al.* Gut microbiota regulates bile acid metabolism by reducing the levels of tauro-beta-muricholic acid, a naturally occurring FXR antagonist. *Cell Metab.* **17**, 225–235 (2013).
92. Wostmann, B. S. Intestinal bile acids and cholesterol absorption in the germfree rat. *J. Nutr.* **103**, 982–990 (1973).
93. Waite, D. W. *et al.* Proposal to reclassify the proteobacterial classes Deltaproteobacteria and Oligoflexia, and the phylum Thermodesulfobacteria into four phyla reflecting major functional capabilities. *International Journal of Systematic and Evolutionary Microbiology* **70**, 5972-6016 (2020).
94. Huerta-Cepas, J. *et al.* eggNOG 4.5: a hierarchical orthology framework with improved functional annotations for eukaryotic, prokaryotic and viral sequences. *Nucleic Acids Res.* **44**, D286–93 (2016).
95. Hausmann, B. *et al.* Peatland Acidobacteria with a dissimilatory sulfur metabolism. *ISME J.* **12**, 1729–1742 (2018).
96. Park, M.-J. *et al.* Establishment of Genome Based Criteria for Classification of the Family Desulfovibrionaceae and Proposal of Two Novel Genera, gen. nov. and gen. nov. *Front. Microbiol.* **13**, 738205 (2022).
97. Laue, H & A M Cook. Biochemical and molecular characterization of taurine:pyruvate aminotransferase from the anaerobe Bilophila wadsworthia. *European journal of biochemistry* **267**, 6841-6848 (2000).
98. Denger, K., Ruff, J., Rein, U. & Cook, A. M. Sulphoacetaldehyde sulpho-lyase (EC 4.4.1.12) from Desulfonispora thiosulfatigenes: purification, properties and primary sequence. *Biochemical Journal* **357**, 581–586 (2001).
99. Laue, H & Cook, A. M. Purification, properties and primary structure of alanine dehydrogenase involved in taurine metabolism in the anaerobe Bilophila wadsworthia. *Archives of microbiology* **174**, 162-167 (2000).
100. Liu, L.-J. *et al.* Thiosulfate transfer mediated by DsrE/TusA homologs from acidothermophilic sulfur-oxidizing archaeon Metallosphaera cuprina. *J. Biol. Chem.* **289**, 26949–26959 (2014).
101. Stockdreher, Y. *et al.* New proteins involved in sulfur trafficking in the cytoplasm of Allochromatium vinosum. *J. Biol. Chem.* **289**, 12390–12403 (2014).
102. Waller, A. S. *et al.* Classification and quantification of bacteriophage taxa in human gut metagenomes.

ISME J. **8**, 1391–1402 (2014).
103. Kieft, K. & Anantharaman, K. Deciphering Active Prophages from Metagenomes. *mSystems* **7**, e0008422 (2022).
