# Supplementary material for: Ecophysiology and interactions of a taurine-respiring bacterium in the mouse gut
Source: Nat Commun. 2023 Sep 18;14:5533. doi: 10.1038/s41467-023-41008-z (PMC10507020; doi:10.1038/s41467-023-41008-z)
Supplement: Supplementary file 3 — Description of Additional Supplementary Files [file 41467_2023_41008_MOESM3_ESM.pdf]

Description of Additional Supplementary Files

Supplementary Data S1. Transcriptome and proteome analyses of *T. muris* LT0009. Columns show the locus tag, gene length in base pairs [bp], transcripts per million [TPM], log fold change, normalized protein expression areas and the annotation. TPM is given as an average of triplicate cultures grown with taurine (T), sulfolactate (SL), and thiosulfate (Thi). In addition, the p-values for the transcripts and protein expressions are provided, with *p*-values <0.01 highlighted in blue and *p*-values <0.05 highlighted in orange.

Supplementary Data S2. 16S rRNA-targeted oligonucleotide probes used for FISH analysis in this study.

Supplementary Data S3. Curated annotation of selected LT0009 genes, with a focus on sulfur and energy metabolism. COG class IDs were assigned by MaGe (Cognitor, www.ncbi.nlm.nih.gov/COG/) and NOG IDs were assigned by the best-match principle [94,95.]

Supplementary Data S4. Prevalence of *Taurinivorans muris* and *Bilophila wadsworthia*-related sequences with 97% identity cut-off across 16S rRNA gene amplicon datasets of diverse hosts. Numbers in parentheses indicate the number of amplicon samples analyzed per host.

Supplementary Data S5. Differential expression of bile salt hydrolase genes in the OMM^{12} community.

Supplementary Data S6. Gene expression of OMM^{12} strains and *S. enterica* Tm SL1344 in gnotobiotic mice with and without LT0009. Gene annotations are derived from the reference genomes at NCBI. log2FC: logarithm to the base2 (Fold Change), padj: adjusted p-value obtained by DESeq2.

Supplementary Data S7. Accession numbers and source information of the sequences used for 16S rRNA phylogenetic analysis, as collected from NCBI (https://www.ncbi.nlm.nih.gov).

Supplementary Data S8. Additional information for *B. wadsworthia*-positive samples from the mouse gut.

Supplementary Data S9. Droplet digital PCR (ddPCR) primers and conditions for quantification of host and Saumur prophage genes in *Enterocloster clostridioformis* YL32.

Supplementary Data S10. Summary of datasets used in this study.
