# Supplementary material for: Ecophysiology and interactions of a taurine-respiring bacterium in the mouse gut
Source: Nat Commun. 2023 Sep 18;14:5533. doi: 10.1038/s41467-023-41008-z (PMC10507020; doi:10.1038/s41467-023-41008-z)
Supplement: Supplementary file 14 — Reporting Summary [file 41467_2023_41008_MOESM14_ESM.pdf]

nature portfolio

Corresponding author(s): Alexander Loy

Last updated by author(s): Aug 11, 2023

Reporting Summary

Nature Portfolio wishes to improve the reproducibility of the work that we publish. This form provides structure for consistency and transparency in reporting. For further information on Nature Portfolio policies, see our Editorial Policies and the Editorial Policy Checklist.

Statistics

For all statistical analyses, confirm that the following items are present in the figure legend, table legend, main text, or Methods section.

n/a	Confirmed	
☐	☒	The exact sample size (*n*) for each experimental group/condition, given as a discrete number and unit of measurement
☐	☒	A statement on whether measurements were taken from distinct samples or whether the same sample was measured repeatedly
☐	☒	The statistical test(s) used AND whether they are one- or two-sided<br>*Only common tests should be described solely by name; describe more complex techniques in the Methods section.*
☒	☐	A description of all covariates tested
☐	☒	A description of any assumptions or corrections, such as tests of normality and adjustment for multiple comparisons
☐	☒	A full description of the statistical parameters including central tendency (e.g. means) or other basic estimates (e.g. regression coefficient) AND variation (e.g. standard deviation) or associated estimates of uncertainty (e.g. confidence intervals)
☐	☒	For null hypothesis testing, the test statistic (e.g. *F*, *t*, *r*) with confidence intervals, effect sizes, degrees of freedom and *P* value noted<br>*Give P values as exact values whenever suitable.*
☒	☐	For Bayesian analysis, information on the choice of priors and Markov chain Monte Carlo settings
☒	☐	For hierarchical and complex designs, identification of the appropriate level for tests and full reporting of outcomes
☒	☐	Estimates of effect sizes (e.g. Cohen's *d*, Pearson's *r*), indicating how they were calculated

Our web collection on statistics for biologists contains articles on many of the points above.

Software and code

Policy information about availability of computer code

Data collection	Detailed in the Materials and Methods or Supplementary Information:<br>daime version 2.2.3<br>Skyline version 22.2 (https://skyline.ms/project/home/software/Skyline/begin.view)<br>LightCycler96 version 1.1
Data analysis	All code used was part of software packages using default parameter if not stated in Method part<br><br>qCAT v. 1.1.0<br>Unicycler v. 0.4.6<br>MUSCLE (v3.8.31)<br>TrimAl (v1.4. rev15)<br>IQ-TREE (v. 1.6.2)<br>HMMER 3.3.2<br>MAFFT (v7.475)<br>FastANI (v. 1.2)<br>Mascot (Matrix Science)<br>Proteome Discoverer v 1.3 (Thermo Fisher Scientific)<br>BBMap (version 37.61, 38.92 and 39.01)<br>PHAST web server (https://phaster.ca/)<br>Deseq2 version 1.36.0

BLASTP web server
R version 4.2.1
iTOL (version 6.7)

For manuscripts utilizing custom algorithms or software that are central to the research but not yet described in published literature, software must be made available to editors and reviewers. We strongly encourage code deposition in a community repository (e.g. GitHub). See the Nature Portfolio guidelines for submitting code & software for further information.

Data

Policy information about availability of data

All manuscripts must include a data availability statement. This statement should provide the following information, where applicable:

- Accession codes, unique identifiers, or web links for publicly available datasets
- A description of any restrictions on data availability
- For clinical datasets or third party data, please ensure that the statement adheres to our policy

Strain LT0009 has been deposited in the German Collection of Microorganisms and Cell Cultures (DSMZ) as DSM111569 and the Japan Collection of Microorganisms (JCM) as JCM34262. The genome and the 16S rRNA gene sequence of T. muris LT0009 are available at NCBI GenBank under accession numbers CP065938 [https://www.ncbi.nlm.nih.gov/nuccore/CP065938.1/] and MW258658 [https://www.ncbi.nlm.nih.gov/nuccore/MW258658.1/], respectively. Sequencing data of the LT0009 pure culture transcriptome (JMF-2012-1) and the mouse gut metatranscriptomes from the HG study (JMF-2101-05) and the gnotobiotic study (JMF-2104-01) were deposited to the NCBI SRA under BioProject accession PRJNA867178 [https://www.ncbi.nlm.nih.gov/bioproject/PRJNA867178]. The mass spectrometry proteomics data have been deposited to the ProteomeXchange Consortium via the PRIDE partner repository with the dataset identifier PXD044449 [https://proteomecentral.proteomexchange.org/cgi/GetDataset?ID=PXD044449]. The LC-MS/MS data for taurine and bile acids quantification is publicly available at the Phaidra repository of the University of Vienna under the persistent identifier 1649944 [https://phaidra.univie.ac.at/o:1649944]. Source data are provided with this paper. All datasets used in this study are summarized in Supplementary Table 10.

GTDB database (release R95, https://gtdb.ecogenomic.org/stats/r95) was used for genome collection. Nucleic acid sequences were collected from the National Center for Biotechnology Information (NCBI https://www.ncbi.nlm.nih.gov/) standard nucleotide database and the SILVA database v.138 (https://www.arb-silva.de/). Protein sequences were obtained from the protein families database (Pfam A https://www.ebi.ac.uk/interpro/download/pfam/).

Research involving human participants, their data, or biological material

Policy information about studies with human participants or human data. See also policy information about sex, gender (identity/presentation), and sexual orientation and race, ethnicity and racism.

Reporting on sex and gender	N/A
Reporting on race, ethnicity, or other socially relevant groupings	N/A
Population characteristics	N/A
Recruitment	N/A
Ethics oversight	N/A

Note that full information on the approval of the study protocol must also be provided in the manuscript.

Field-specific reporting

Please select the one below that is the best fit for your research. If you are not sure, read the appropriate sections before making your selection.

☒ Life sciences ☐ Behavioural & social sciences ☐ Ecological, evolutionary & environmental sciences

For a reference copy of the document with all sections, see nature.com/documents/nr-reporting-summary-flat.pdf

Life sciences study design

All studies must disclose on these points even when the disclosure is negative.

Sample size	No statistical methods were used to pre-determine sample size. For all growth experiments, we performed triplicates. We sampled all individuals in the animal experiments (see below). The numbers were chosen based on feasibility and to comply with minimal replication requirements for statistical analyses.
Data exclusions	No data was excluded
Replication	The growth experiments with the isolated strain were performed repeatedly and same growth conditions were used for downstream analysis. Optimal growth conditions, e.g. temperature, pH, taurine concentrations, electron donors, and electron acceptors, were performed in

	triplicates, but tested only once. Growth experiments for transcriptome and proteome were performed once in triplicates and sampled at the same growth phase. The gnotobiotic Oligo-Mouse-Microbiota mouse experiment was performed once with n=6 mice for each of the two groups. Mono-/co-colonization mouse experiment was performed once with 7 mice and samples were collected at different time points. High-glucose diet experiment in mice was performed once with n=3 mice for each of the two groups. All attempts at replication were successful and no data were excluded for analysis.
Randomization	We ensured gender balance within the experimental groups by incorporating an relative equal number of male and female mice in OMM12 mouse and high-glucose diet experiments. Only female mice were used for mono-/ co-colonization experiment, thus mice were assigned to different groups randomly.
Blinding	Blinding of samples/data was not performed as no subjective scoring was performed in this study.

Reporting for specific materials, systems and methods

We require information from authors about some types of materials, experimental systems and methods used in many studies. Here, indicate whether each material, system or method listed is relevant to your study. If you are not sure if a list item applies to your research, read the appropriate section before selecting a response.

Materials & experimental systems

n/a	Involved in the study
☒	☐ Antibodies
☒	☐ Eukaryotic cell lines
☒	☐ Palaeontology and archaeology
☐	☒ Animals and other organisms
☒	☐ Clinical data
☒	☐ Dual use research of concern
☒	☐ Plants

Methods

n/a	Involved in the study
☒	☐ ChIP-seq
☒	☐ Flow cytometry
☒	☐ MRI-based neuroimaging

Animals and other research organisms

Policy information about studies involving animals; ARRIVE guidelines recommended for reporting animal research, and Sex and Gender in Research

Laboratory animals	C57BL/6 mice aged 7-20 weeks
Wild animals	No wild animals were used in this study
Reporting on sex	We ensured gender balance within the experimental groups by incorporating an relative equal number of male and female mice in the OMM12 mouse experiment. Only female mice were used for mono-/ co-colonization and high-glucose diet experiments.
Field-collected samples	No field-collected samples
Ethics oversight	Animal experiments were approved by the local authorities in Germany (Regierung von Oberbayern; ROB-55.2-2532.Vet_02-20-84) or by national Austrian authorities (BMWF-66.006/ 0032-WF/V/3b/2014).

Note that full information on the approval of the study protocol must also be provided in the manuscript.
